# Integrating perfluorocarbon and type-I photosensitizer into a single molecule: a phenothiazine-based platform for hypoxia-tolerant PDT

**DOI:** 10.1039/d6ra05336j

**Published:** 2026-08-12

**Authors:** Ziqi Zou, Meiying Liu, Kun Zhang, Peng Luo, Tongsheng Huang, Jianwen Tian, Xiaoyong Zhang, Zhihui Kuang, Yen Wei

**Affiliations:** a Orthopedic Hospital, The First Affiliated Hospital, Jiangxi Medical College, Nanchang University No. 17 Yong Wai Zheng Street Nanchang Jiangxi 330006 China kuangzhihui@email.ncu.edu.cn; b Department of Chemistry, Nanchang University 999 Xuefu Avenue Nanchang Jiangxi 330031 China zhangxiaoyong@ncu.edu.cn tjwen820@126.com; c Key Laboratory of Modern Preparation of TCM, Ministry of Education, Jiangxi University of Traditional Chinese Medicine Nanchang 330004 China; d Department of Chemistry and the Tsinghua Center for Frontier Polymer Research, Tsinghua University Beijing 100084 P. R. China

## Abstract

Hypoxia-induced resistance remains a major obstacle in photodynamic therapy (PDT) due to the oxygen dependency of conventional type-II photosensitizers. To tackle this issue, we developed a phenothiazine-based small molecule (PFI) that integrates intrinsic oxygen-carrying perfluoroalkyl chains with type-I photodynamic functionality. The perfluorinated segments enable localized oxygen enrichment to counteract tumor hypoxia, while the phenothiazine core generates reactive oxygen species (ROS) *via* an oxygen-independent type-I pathway, sustaining phototoxicity even under low-oxygen environments. Both *in vitro* and *in vivo* studies confirm that PFI effectively accumulates in tumors, relieves hypoxia, and achieves potent antitumor efficacy with negligible side effects. This single-component design offers a facile and robust platform for hypoxia-tolerant photodynamic cancer therapy.

## Abstract 2

A single-molecule platform integrating a perfluorinated oxygen carrier with a type I photosensitizer overcomes tumor hypoxia for enhanced photodynamic therapy.

## Graphical abstract

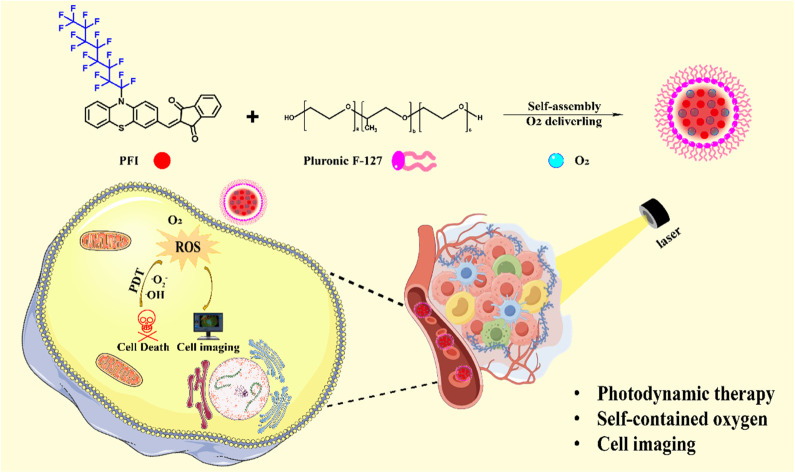

## Introduction

Tumor hypoxia is a pervasive hallmark of solid tumors, with oxygen partial pressures frequently falling below the threshold required for effective oxygen-dependent therapies.^1^ This pathological condition not only directly compromises the efficacy of photodynamic therapy (PDT) by limiting the generation of reactive oxygen species (ROS), but also promotes immunosuppression, metastasis, and therapeutic resistance. Conventional type-II PDT relies on molecular oxygen to generate singlet oxygen (^1^O_2_), making it particularly vulnerable to hypoxic microenvironments.^2^ Moreover, PDT itself consumes local oxygen during illumination, potentially exacerbating tumor hypoxia and creating a vicious cycle that undermines treatment outcomes and may even drive tumor progression.^3^

**Scheme 1:**
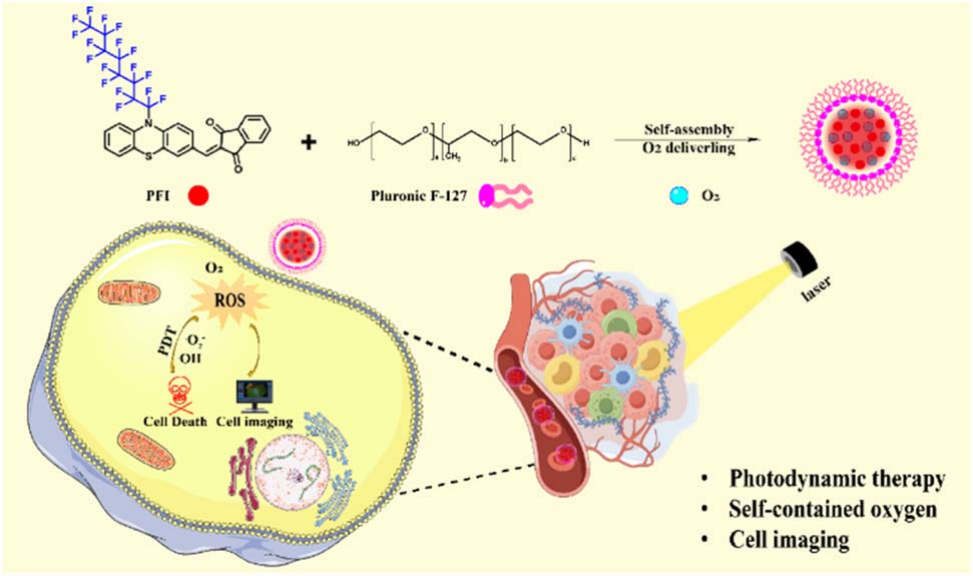
Synthetic route of PFI and preparation of PFI NPs for cellular imaging and PDT.

Extensive efforts have been devoted to mitigating tumor hypoxia through two principal strategies. The first aims to increase local oxygen availability using exogenous oxygen carriers, *in situ* oxygen generation *via* catalase-like nanozymes or hydrogen peroxide decomposition, and hypoxia-activated photosensitizers (PSs).^4^ The second strategy pursues oxygen-independent PDT modalities, particularly Type-I PSs that generate superoxide anion (˙O_2_^−^) and hydroxyl radicals (˙OH) through electron transfer mechanisms independent of molecular oxygen.^5^ Among oxygen carriers, perfluorocarbons (PFCs) are particularly attractive oxygen carriers due to their high oxygen solubility, inertness, and biocompatibility.^6^ This has led to various PFC-based nanoplatforms designed to relieve tumor hypoxia and enhance PDT. However, current systems typically rely on the physical encapsulation or co-assembly of separate PFCs and PSs. These multi-component systems are plagued by inherent drawbacks, including batch-to-batch variability and complex assembly.^7^ Accordingly, a more elegant molecular design that unites oxygen-carrying and photosensitizing functions within a single, well-defined entity is urgently needed to achieve synchronized delivery and coordinated therapeutic action at the tumor site. In this contribution, we report a streamlined molecular strategy that integrates intrinsic oxygen self-carrying capacity with efficient type I photosensitization within a single phenothiazine (PHE)-based small molecule (PFI), in which the perfluorinated segment functions as an oxygen reservoir, while the donor–π–acceptor (D–π–A) framework enables type I photosensitization (Scheme 1). This single-component design ensures stoichiometric fidelity, synchronized delivery to cancer cells, and coordinated oxygen delivery upon photoactivation. By circumventing the need for multi-component formulations, this work establishes a new paradigm for the design of hypoxia-tolerant photodynamic agents, offering a promising therapeutic strategy to enhance PDT efficacy.

## Results and discussion

**Fig. 1:**
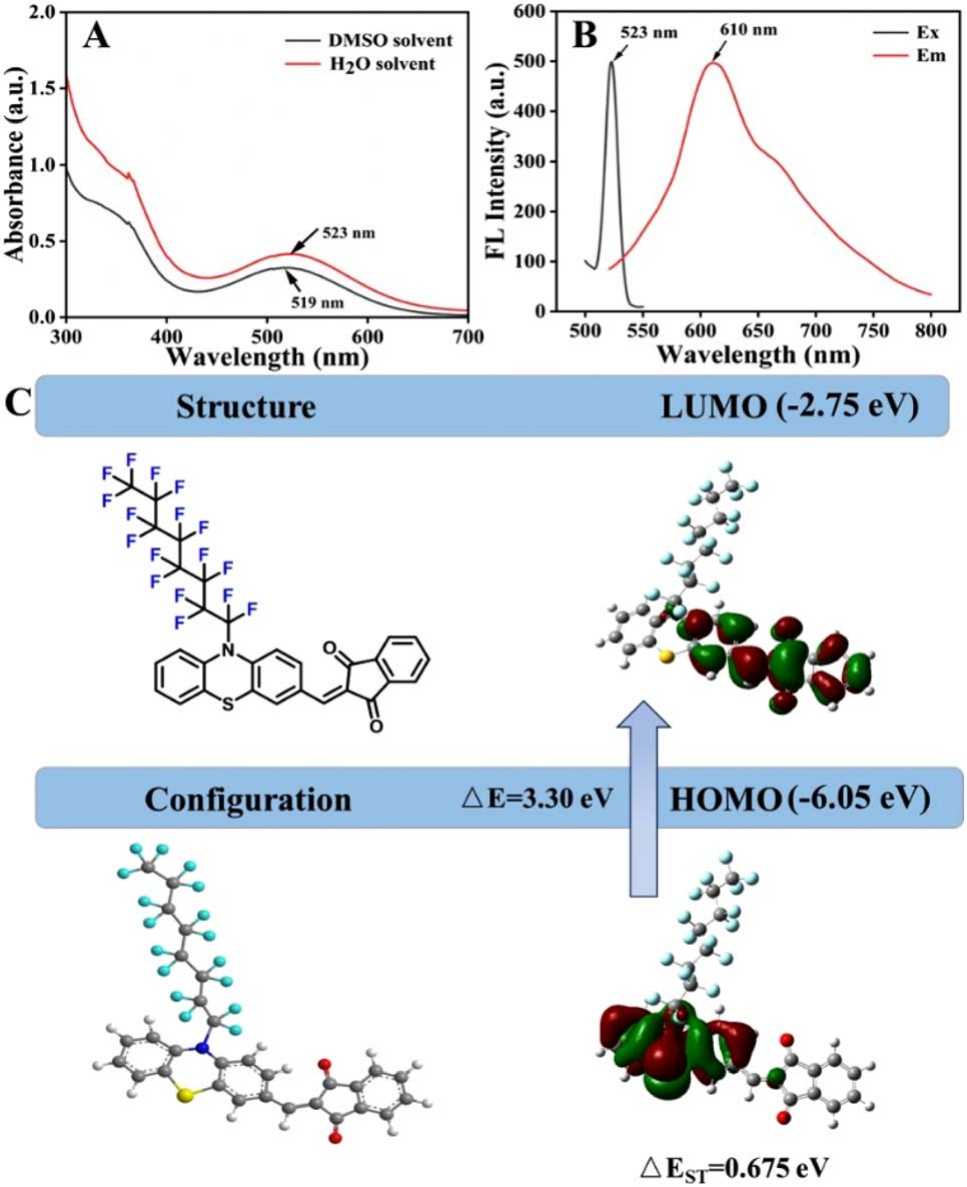
(A) UV absorption spectra of PFI in water and DMSO, respectively; (B) excitation and emission spectra of PFI; (C) molecular structure, configuration, HOMO and LUMO of PFI using Gaussian 16W (B3LYP/6-311+G).

The target molecule PFI was synthesized *via* a three-step procedure (Scheme S1). A perfluorooctyl chain was first introduced onto the PHE scaffold *via* nucleophilic substitution, followed by Knoevenagel condensation with 1,3-indandione to construct the D–π–A framework. All intermediates and the final product were fully characterized by NMR spectroscopy (Fig. S1–S6). Successful grafting of the perfluoroalkyl chain was confirmed by characteristic ^19^F NMR signals at −80 ppm (–CF_3_) and −126 ppm (–CF_2_–), while ^1^H and ^13^C NMR analysis verified the stepwise introduction and consumption of the formyl group, corroborating the formation of PFI (Fig. S7 and S8). PFI exhibits a broad intramolecular charge transfer (ICT) absorption band (500–550 nm) and emission at 610 nm (Fig. 1A and B). The large Stokes shift suggests significant excited-state relaxation, which is beneficial for minimizing self-quenching. The absorption and emission profiles remained similar in DMSO and water, indicating minimal perturbation by aqueous environments. Solvent-dependent studies showed systematic blue-shifting of the ICT band with decreasing solvent polarity, further confirming the ICT character of the visible transition (Fig. S9 and S10). The significant ICT characteristics not only confer a large Stokes shift on PFI (which is beneficial for fluorescence imaging and reduces self-absorption), but also play a crucial foundation for efficient charge separation and subsequent electron transfer (type-I photosensitization process). To further elucidate the electronic structure, time-dependent density functional theory (TD-DFT) calculations were performed at the B3LYP/6-311+G level. The highest occupied molecular orbital (HOMO) is localized on the electron-donating PHE core, while the lowest unoccupied molecular orbital (LUMO) resides on the indandione acceptor (Fig. 1C). The calculated HOMO–LUMO gap (Δ*E*_ST_ = 3.30 eV) is consistent with the observed green-region absorption and corroborates the ICT character. This spatial orbital separation not only rationalizes the large Stokes shift but also suggests efficient charge separation upon photoexcitation. Collectively, these results establish PFI as a structurally integrated, single-component platform that combines oxygen self-carrying capacity with Type I photosensitization, offering a streamlined approach to overcome hypoxia in PDT.

**Fig. 2:**
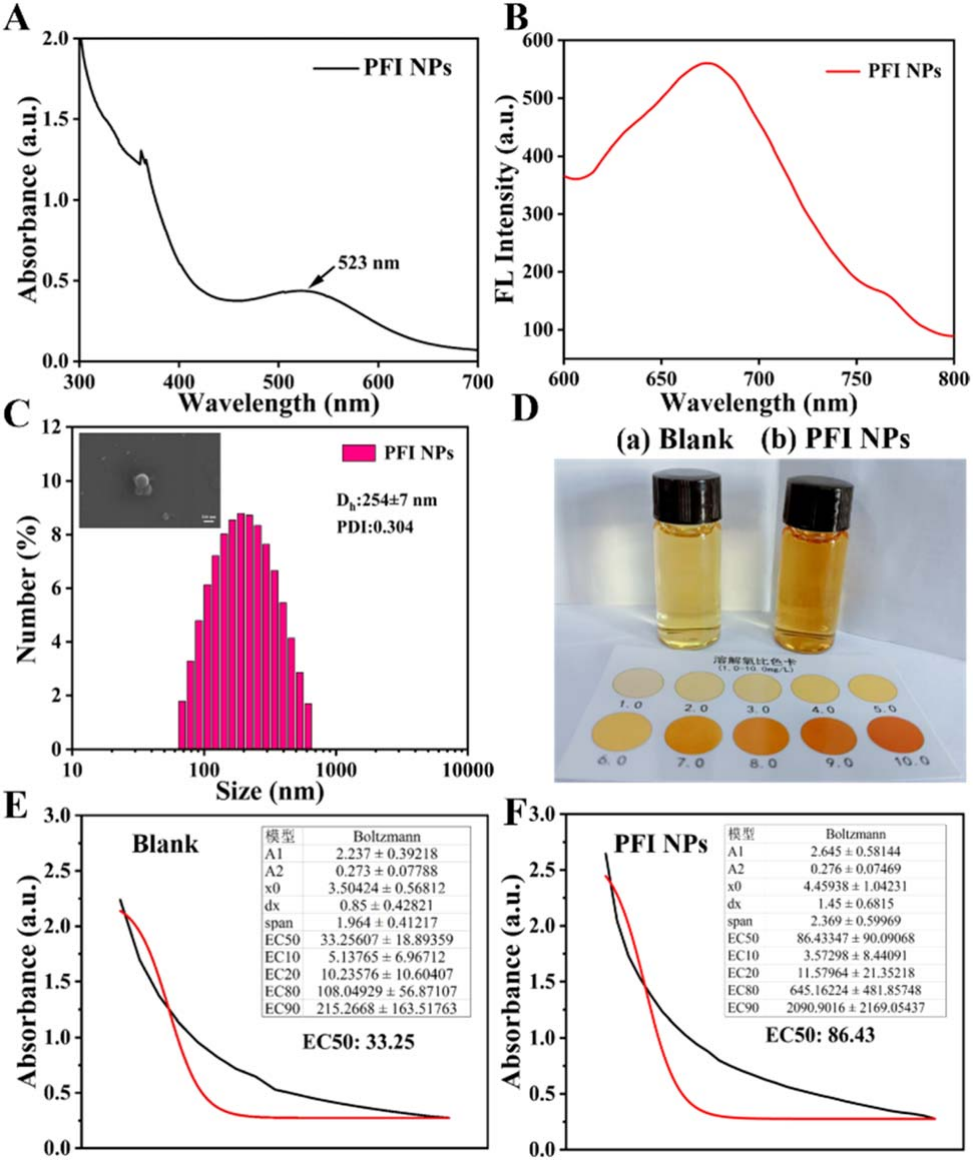
(A) UV absorption spectrum of PFI NPs in pure water; (B) fluorescence emission spectrum of PFI NPs in pure water; (C) size distribution analysis of PFI NPs by dynamic light scattering (DLS); (D) colorimetric dissolved oxygen (DO) assay. A visual comparison between pure water (left) and PFI NPs suspension (right) after treatment with DO indicator reagent; (E and F) after diluting the sample gradient, fit the obtained data to a curve by measuring absorbance at 287 nm, analyze and compare EC_50_ values (the EC_50_ represents the half-maximal effective concentration of oxygen consumption in the indicator reaction system) to assess oxygen demand.

To enhance its aqueous dispersibility, PFI nanoparticles (PFI NPs) were prepared *via* co-assembly PFI with Pluronic F127. The PFI NPs exhibit an absorption maximum at 523 nm and red emission (650–700 nm) in water (Fig. 2A and B). As evidenced by scanning electron microscope (SEM) images, PFI NPs show uniform spherical morphology with a dry diameter of ∼150 nm (Fig. S11). Corresponding energy dispersive X-ray spectroscopy (EDS) mapping confirms homogeneous distribution of C, N, O, F, and S; the strong fluorine signal (20.66 at%) verifies perfluorooctyl chain incorporation without phase separation (Fig. S12). Dynamic light scattering (DLS) measurement gives a hydrodynamic diameter of 254 ± 7 nm (PDI = 0.304) (Fig. 2C), which is well consistent with SEM results. The colloidal stability of PFI nanoparticles was evaluated by monitoring their hydrodynamic size and PDI in water and PBS over 7 days (Fig. S13). The particle size remained stable within 315–355 nm in water and 320–355 nm in PBS, with consistently low PDI values throughout, indicating excellent size distribution uniformity. Additionally, exposure to white light (50 mW cm^−2^) for up to 60 min caused no significant changes in either size or PDI (Fig. S14). A zeta potential of −51.7 mV implies its high colloidal stability (Fig. S15). More importantly, PFI NPs exhibit superior oxygen-carrying capacity, with dissolved oxygen levels of 7–8 mg L^−1^*versus* 4–5 mg L^−1^ for water (Fig. 2D). Fitting absorbance data at 287 nm yields an EC_50_ of 86.43 for PFI NPs, significantly higher than 33.25 for water, confirming enhanced oxygen loading (Fig. 2E and F). The higher EC_50_ value for PFI NPs compared to pure water indicates that PFI NPs can sustain a higher oxygen load before reaching the half-saturation point of the indicator, reflecting their superior oxygen-carrying capacity (approximately 2.6-fold enhancement in oxygen loading).

**Fig. 3:**
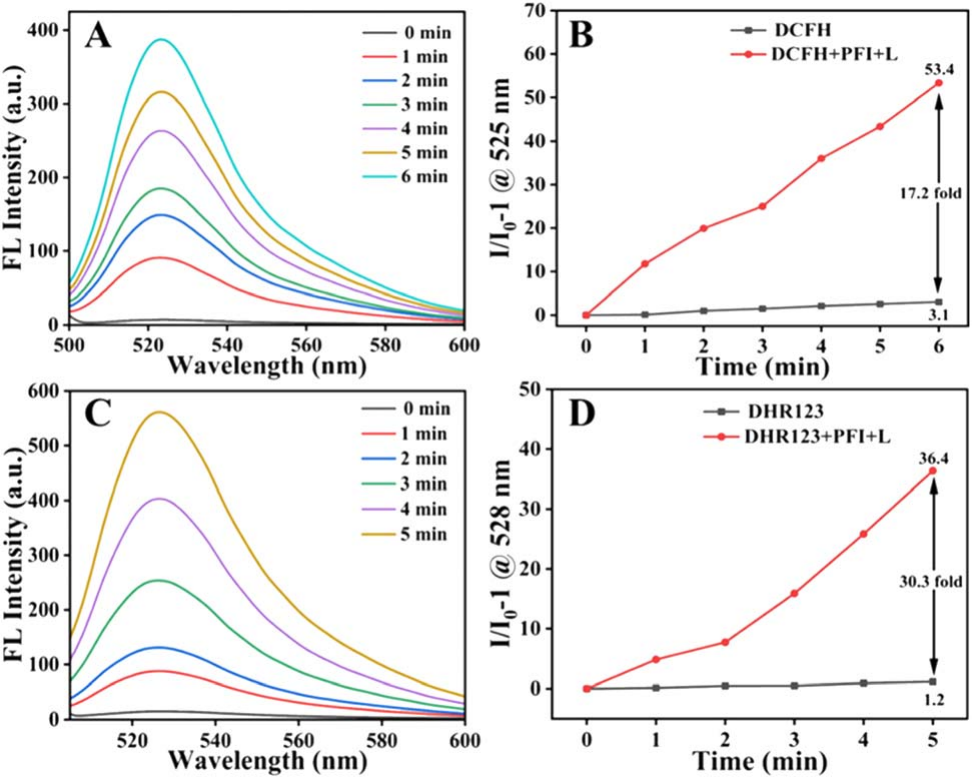
ROS generation by PFI under white light irradiation (50 mW cm^−2^). (A) Time-dependent DCFH fluorescence spectra showing rapid increase upon PFI-mediated photoactivation. (B) Kinetic traces of DCFH fluorescence at 525 nm. (C) DHR123 fluorescence spectra confirming efficient ˙O_2_^−^ generation. (D) Corresponding DHR123 fluorescence intensity at 528 nm.

Using DCFH-DA as a general ROS probe, rapid fluorescence enhancement at 525 nm was observed upon white light irradiation, reaching a 17.2-fold increase after 6 min (Fig. 3A and B). Control experiments without PFI showed negligible signal change (Fig. S16). ABDA assays revealed no detectable singlet oxygen (^1^O_2_) production (Fig. S17), ruling out a type II mechanism. In contrast, DHR123 probing confirmed efficient ˙O_2_^−^ generation, with a 30.3-fold fluorescence increase after 5 min irradiation (Fig. 3C and D). These results establish PFI as a major type I PS generating ˙O_2_^−^ as the primary ROS species. PFI NPs retained this photodynamic activity, exhibiting substantial ROS production upon irradiation with ˙O_2_^−^ as the dominant species (Fig. S18–S20), confirming that nanoformulation does not compromise the intrinsic photofunction of PFI. Furthermore, our previous studies established that PHE-based PSs exhibit aggregation-enhanced ROS generation, making nanoassembly a strategic necessity rather than merely a formulation requirement.^8^ Building on this principle, PFI embodies a paradigm shift in PS design. We moved beyond conventional optimization of the D–A framework by introducing a perfluoroalkyl chain onto the PHE nitrogen. This strategic modification confers intrinsic oxygen-carrying capacity, directly tackling the oxygen dependency that hampers conventional PDT. The resulting integration of reversible oxygen storage with Type I photosensitization within a single molecular scaffold provides a streamlined strategy to overcome tumor hypoxia.

**Fig. 4:**
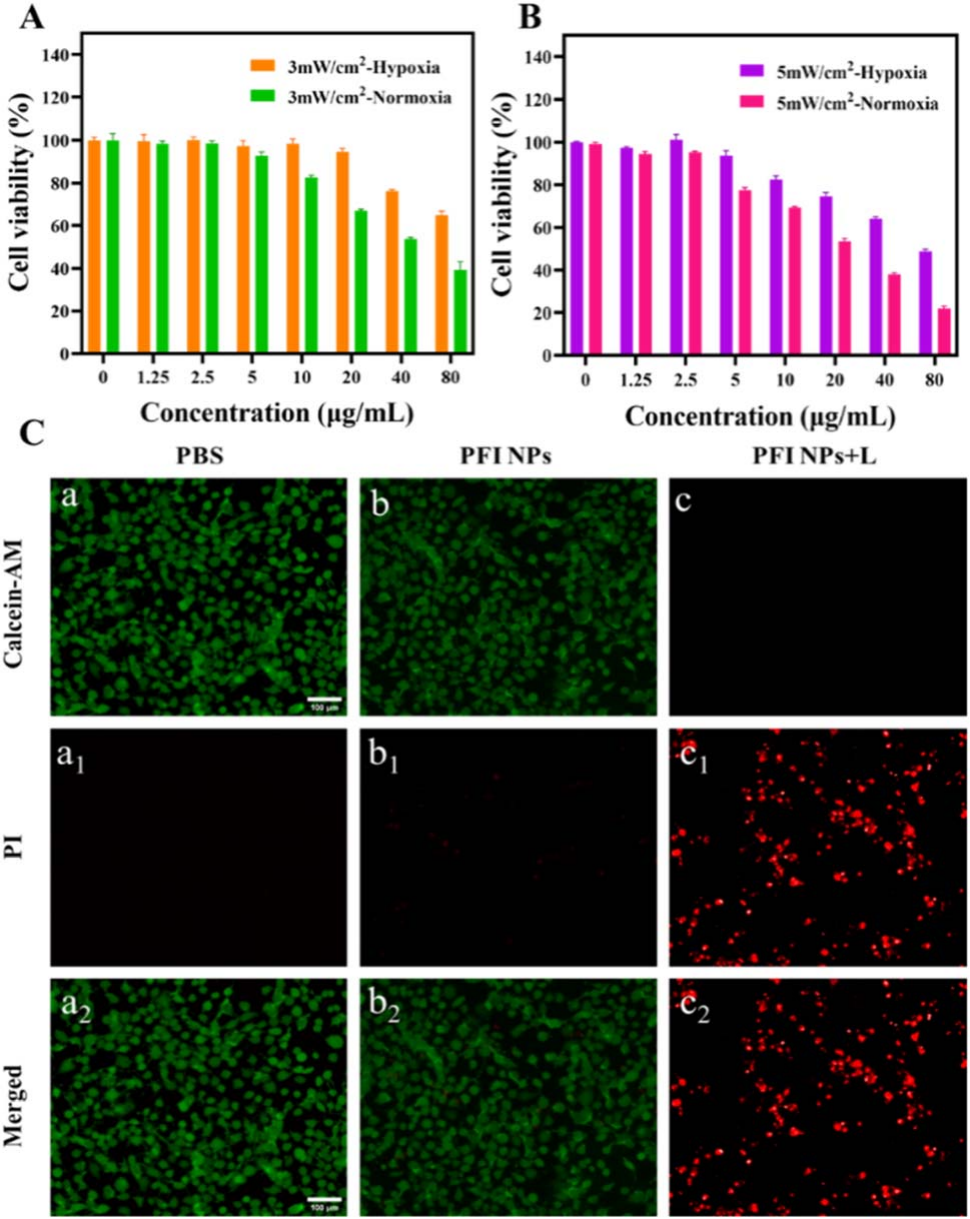
Cell viability of MDA-MB-231 cells co-incubated with PFI NPs at different concentration gradients for 24 h upon irradiation under normoxic (21% O_2_) or hypoxic (with 50 µM CoCl_2_) conditions (*n* = 3) (A) 3 mW cm^2^; (B) 5 mW cm^−2^. (C) Co-stained inverted fluorescence images of PFI NPs and Calcein-AM/PI after incubation in MDA-Mb-231 breast cancer cells followed by 3 min illumination (A: PBS; B: No light exposure; C: 3 min of light exposure). White light intensity: 5 mW cm^2^; measured concentration: 80 µg mL; scale bar = 100 µm.

To determine the potential of PFI NPs for biomedical applications, cellular internalization of PFI NPs was examined by confocal microscopy. As shown in Fig. S21, distinct red fluorescence was observed in the cytoplasm, clearly separated from DAPI-stained nuclei after 4 h incubation, confirming efficient cellular uptake. This cytoplasmic localization positions the PS in proximity to key organelles, maximizing photodamage potential. Intracellular ROS generation was then verified. Using DCFH-DA as the probe, strong green fluorescence appeared only in cells treated with PFI NPs and light; controls showed negligible signals (Fig. S22). DHE staining, specific for ˙O_2_^−^, revealed intense red fluorescence exclusively under PFI NPs plus light irradiation (Fig. S23), confirming that PFI NPs retain their type I photodynamic mechanism within cells. *In vitro* cytotoxicity was evaluated using the CCK-8 assay. PFI NPs exhibited minimal dark toxicity under normoxia, with >80% cell viability even at 80 µg mL^−1^ (Fig. S24), indicating excellent biocompatibility. A chemically induced hypoxia model was established using CoCl_2_; 50 µM was identified as the optimal concentration, inducing hypoxia with minimal cytotoxicity (Fig. S25).^9^ Under light irradiation (3 mW cm^−2^ and 5 mW cm^−2^), PFI NPs showed dose-dependent photocytotoxicity under both normoxic and hypoxic conditions (Fig. 4A and B). As expected, antitumor efficacy was slightly reduced under hypoxia, indirectly corroborating the oxygen-carrying function of PFI NPs. Notably, at 80 µg mL^−1^ with light exposure, the cell survival rate dropped to about 25%, demonstrating potent photodynamic activity. Live/dead staining using calcein-AM/PI provided visual confirmation (Fig. 4C). Controls (PBS with light, PFI NPs alone) showed predominantly green fluorescence (live cells). In contrast, PFI NPs plus light yielded extensive red fluorescence (dead cells) with minimal green signal, directly visualizing the potent photodynamic killing effect.

## Conclusions

In summary, we have developed a PHE-based PS (PFI) that covalently integrates a perfluorinated oxygen-carrying chain with a type I photodynamic core. This single-molecule design yields nanoparticles capable of efficient ˙O_2_^−^ generation and intrinsic oxygen supply, directly addressing the dual challenges of PS performance and tumor hypoxia in PDT. This work establishes a generalizable strategy for hypoxia-tolerant phototherapeutics, with modular design enabling tunable properties and future multifunctional applications, providing a foundation for next-generation PDT agents that actively modulate the tumor microenvironment.

## Conflicts of interest

There are no conflicts to declare.

## Supplementary Material

RA-OLF-D6RA05336J-s001

## Data Availability

The data supporting this article have been included as part of the supplementary information (SI). Supplementary information: the ^1^H NMR, ^19^F NMR, and ROS generation results of samples *et al.* were shown in SI. See DOI: https://doi.org/10.1039/d6ra05336j.
