# Supplementary material for: Integrating perfluorocarbon and type-I photosensitizer into a single molecule: a phenothiazine-based platform for hypoxia-tolerant PDT
Source: RSC Adv. 2026 Aug 12;16(40):47133–7. doi: 10.1039/d6ra05336j (PMC13463296; doi:10.1039/d6ra05336j)
Supplement: Supplementary file 1 [file RA-016-D6RA05336J-s001.pdf]

Supplementary Information (SI) for RSC Advances.
This journal is © The Royal Society of Chemistry 2026

Supporting Information

Integrating Perfluorocarbon and Type-I Photosensitizer into a Single Molecule: A Phenothiazine-Based Platform for Hypoxia-Tolerant PDT

Ziqi Zou[1,2,#]; Meiying Liu[2,3,#], Kun Zhang[1,2]; Peng Luo[1,2], Tongsheng Huang[1,3]; Jianwen Tian[2,*]; Xiaoyong Zhang[2,*]; Zhihui Kuang[1,*]; Yen Wei[4]

[1] Orthopedic Hospital, the First Affiliated Hospital, Jiangxi Medical College, Nanchang University, No. 17 Yong Wai Zheng Street, Nanchang, Jiangxi 330006, China.

[2] Department of Chemistry, Nanchang University, 999 Xuefu Avenue, Nanchang, Jiangxi 330031, China.

[3] Key Laboratory of Modern Preparation of TCM, Ministry of Education, Jiangxi University of Traditional Chinese Medicine, Nanchang 330004, China.

[4] Department of Chemistry and the Tsinghua Center for Frontier Polymer Research,

Tsinghua University, Beijing, 100084, P. R. China.

Email address: zhangxiaoyong@ncu.edu.cn

1 Experimental section

1.1 Materials and measurements

Phenothiazine (PHE), Perfluoro-n-Octyl Iodide, 1,3 indandione（IN）were purchased from Adamas. Dihydrorhodamine 123 (DHR123), 9,10-anthracenediyl-bis(methylene)-dipropanedioic acid (ABDA), 2,7-dichlorodihydroluciferin diacetate (H_2DCF-DA) were supplied by Aladdin. All other reagents were obtained from commercial sources and were used as received without additional purification, unless otherwise specified. ^{19}F NMR, ^{1}H NMR and ^{13}C NMR spectra were measured on a Bruker Avance-400 spectrometer at room temperature. UV-visible absorption spectra were measured on a UV-1700 spectrophotometer. UV-visible absorption spectra were measured on a UV-1700 spectrophotometer. Fluorescence spectra were recorded on a F97 Fluorospectrophotometer. Fluorescence cell images were performed on a fluorescence inverted microscope (Ts2R, Japan) and a confocal laser scanning microscope (CLSM, Dragonfly CR-DFLY-505, Andor).

1.2 Synthesis and characterization of compounds 1-2 and PFI

The compound 1-2 and target products were synthesized according to the procedures as shown in **Scheme S1**. The compounds 1 were synthesized through Nucleophilic substitution reaction (**Scheme S1**). The compounds 2 were acquired by Vilsmeier-Haack reaction. The target products were obtained by Knoevenagel reaction of compounds 2 with 1,3 indandione (IN). The chemical structures of compound 1 was characterized by ^{1}H NMR and ^{19}F NMR spectroscopy and target product was characterized by ^{1}H NMR and ^{13}C NMR spectroscopy and these spectra were provided in **Fig. S1-8**.

1.2.1 Synthesis of compound 1

Phenothiazine (7.2 g, 36 mmol) and NaH (14.4 g) were placed in an ice bath and dissolved in anhydrous DMF (60 mL), followed by addition of Perfluoro-n-Octyl Iodide (19.8 g, 36 mmol). The mixture reacted at room temperature for 24 h. Spot analysis indicated near-complete disappearance of the starting material spot, indicating reaction completion. Excess water was added, followed by extraction with dichloromethane (30 mL × 6) and six water washes. The organic mixture was dried over anhydrous sodium sulfate, and purified by column chromatography using petroleum ether/ethyl acetate (5:1, v/v) as the eluent to afford a deep green powdery solid of perfluorinated PHE. Yield: 50 %. ^{1}H NMR (400 MHz, $CDCl_3$) δ 7.23 (s, 2H), 6.95 (s, 2H), 6.80 (s, 2H), 6.23 (s, 2H). ^{19}F NMR (376 MHz,

$CDCl_3$) δ -80.83, -122.00 (t, J = 271.3 Hz), -126.17.

1.2.2 Synthesis of compound 2

DMF (2.6 mL, 6.7 mmol) and 1,2-dichloroethane (8 mL) were stirred at 0°C for 10 minutes. $POCl_3$ (2.5 mL, 5 mmol) was injected into the reaction flask, and perfluorinated PHE (3.09 g, 5 mmol) dissolved in 1,2-dichloroethane (8 mL) was injected into the reaction flask. The mixture was refluxed under nitrogen for 12 hours overnight. The product exhibited fluorescence, and the reaction was monitored by plate tracking. Upon reaction completion, add sodium bicarbonate aqueous solution to neutralize acidic gases in the reaction mixture (bubbles will form; add slowly). The mixture was extracted with dichloromethane, dried over anhydrous sodium sulfate, and purified by column chromatography using petroleum ether/ethyl acetate (2:1, v/v) as the eluent. The resulting compound 2 was a yellow powdery solid. Yield: 54 %. ^{1}H NMR (400 MHz, $CDCl_3$) δ 9.68 (s, 1H), 8.00 (s, 1H), 7.42 (s, 1H), 7.25 (s, 1H), 6.92 (s, 1H), 6.83 (s, 1H), 6.47 (s, 1H), 6.23 (s, 1H).

1.2.3 Synthesis of PFI

Perfluoro-PHE-CHO (617 mg, 1 mmol) and 1,3-indandione (175 mg, 1.2 mmol) were dissolved in chloroform (20 mL), and pyridine (0.5 mL) was added. The mixture was refluxed overnight, spotted onto a plate, and purified by column chromatography using petroleum ether/ethyl acetate (1:1, v/v) as the eluent to afford the red powdery solid PFI. Yield: 58 %. ^{1}H NMR (400 MHz, $CDCl_3$) δ 8.87 (s, 1H), 7.96 (d, J = 26.4 Hz, 1H), 7.81 – 7.66 (m, 1H), 7.64 – 7.47 (m, 1H), 7.25 (s, 1H), 7.13 (t, J = 7.4 Hz, 1H), 7.00 (t, J = 7.8 Hz, 1H), 6.94 (s, 1H), 6.83 (s, 2H), 6.22 (d, J = 7.5 Hz, 2H). ^{13}C NMR (101 MHz, $CDCl_3$) δ 137.16, 130.87, 129.87, 128.77, 124.14, 123.58, 120.49, 68.13.

1.3 Preparation of PFI NPs

5 mg of PFI and 15 mg of F-127 were dissolved completely in 2 mL of THF to form a clear solution. Separately, place 6 mL of 0.1 M phosphate buffer solution (pH = 7.4) in a centrifuge tube and subject it to ultrasonication at maximum power for 1 minute. While maintaining continuous ultrasonication of the buffer, add the aforementioned THF solution dropwise over approximately 1 min. Subsequently, continue ultrasonicating the mixture for 10 min, then remove the THF via rotary evaporation. Finally, subject the mixture to freeze-drying to obtain a dry nanoparticle solid.

1.4 ROS detection

1.4.1 Total ROS detection

Measurement of ROS produced by 20 μL PS (50 μg/mL) and 4 mL H_2DCF-DA (1 μM) were added to a cuvette. Then, the cuvette was irradiated with a white light source (50 mW/cm^2) for different times and the PL spectra were measured from 500 to 650 nm.

1.4.2 Singlet oxygen detection

The $^1O_2$ generated by PSs after light triggering was measured using ABDA as the indicator. 30 μL PS (1 mg/ml) and 45 μL ABDA (4 mM) were added to a cuvette containing PBS solution (2925 μL). The cuvette was then irradiated with a white light source (50 mW/cm^2) for different times and absorption spectra were collected from 340 to 420 nm.

1.4.3 Superoxide anion radical detection

Herein, superoxide anion radicals ($\cdot O_2^-$) produced by PSs were detected by combining dihydrorhodamine 123 (DHR 123). First, 15 μL PSs (0.2 mg/ml) and 15 μL DHR123 (0.2 mM) were added to a cuvette containing PBS solution (2970 μL). Then, the cuvette was irradiated with a white light source (50 mW/cm^2) for different times and the PL spectra were measured from 500 to 650 nm.

1.5 photodynamic experiments

We selected MDA-MB-231 cells as a model to measure intracellular ROS production with DCFH-DA under light conditions. The cells were seeded into 6-well plates at a density of 2 × 10^6 cells/well, incubated at constant temperature for 24 h, and then treated with the prepared sample at the desired concentration for 6 h. The medium was then aspirated, and the cells were gently washed three times with PBS. Subsequently, 20 μM DCFH-DA and 20 μM DHE fluorescent probes were added to fresh medium, and the cells were incubated at 37 °C for 30 min. After incubation, the probes were removed by rinsing the cells again with PBS. Finally, PBS buffer solution was added into the well plates to infiltrate the cells, and then the plates were placed in a laser with a light intensity of 5 mW/cm^2 and irradiated for 3 min at 450 nm (energy dose: 0.9 J/cm^2), and then the cells were inverted and the fluorescence was taken under an inverted fluorescence microscope.

1.6 Cellular uptake and fluorescence imaging

First, MDA-MB-231 breast cancer cells were obtained from the Cell Bank of the Typical Culture Collection Committee, Chinese Academy of Sciences (TCCC, CAS), Shanghai, China. The cells were plated at a density of 1.0 × 10^6 cells/well at six-well plates before experimental manipulation and

incubated in a constant temperature incubator at 37°C for one day to achieve a growth density of about 80%. Then 30 μg/mL of PFI NPs were added to each well and incubated for 8 h under dark conditions and then washed with PBS buffer solution for three times to remove the residual samples, followed by the addition of 1 mL of Hoechst 33342 stain to the six-well plate, and the incubation was continued for half an hour in a thermostatic incubator, and then, the stain and the medium were aspirated from the six-well plate and washed with PBS for 2-3 times to remove the staining agent so that the background would not be too bright and affect the photographic effect. Finally, the fluorescence intensity was observed under an integrated fluorescence inverted microscope and the fluorescence images under the corresponding channels were taken.

1.7 Cell viability

To evaluate the effects of PFI NPs on the viability of MDA-MB-231 cells, the specific operation was to inoculate MDA-MB-231 into 96-well plates at a cell density of 5×10^3 cells/well, and then place them in an incubator for 24 h. Then the cells were treated with different concentrations of the test samples, and the incubation was continued for 24 h (normoxic conditions). Under hypoxic conditions, cells were first treated with cobalt chloride ($CoCl_2$) for 4 hours to establish hypoxia, each group of cells was treated with 50μM $CoCl_2$, followed by treatment with test samples at varying concentrations [1-3]. The CCK-8 kit was used to test cell viability, the experimental procedure was to remove the medium, add 100 μL of freshly prepared CCK-8 solution (0.5 mg/mL) to each well, and continue the incubation for 2 h under the condition of avoiding light. The absorbance of each well was measured at 450 nm using an enzyme labeling instrument, and the calculated results were expressed as the mean ± standard deviation. The experimental procedure for phototoxicity can be referred to darkness. The first step was to perform cell inoculation with cell inoculation density and incubation conditions consistent with those used in the dark toxicity experiments. Subsequently, the cells were treated with different concentrations of sample solutions for a duration of 8 h. After treatment, the cells were exposed to a 450 nm laser with an intensity of 3 mW/cm^2 and 5 mW/cm^2 for 3 min. After the laser irradiation, the cells continued to be incubated for 16 h. Next, the CCK-8 reagent was added to the 96-well plate and incubated for 2 h. Finally, cell activity was detected using a microplate reader at an absorption wavelength of 450 nm. The results were expressed as mean ± standard deviation.

1.8 Live-dead cell staining

MDA-MB-231 cells were inoculated into 6-well plates at a density of 4×10^5 cells/well per well and

incubated for 24 h. Cells were then treated with 80 μg/mL of sample solution and incubated under dark conditions for 4 h. Subsequently, cells were exposed to a 450 nm laser with an intensity of 5 mW/cm^2 for 3 min. After laser irradiation, cells were washed with PBS buffer solution three times to remove the residual sample solution. Next, 2 μM of Calcein-AM was added to each well and incubated for 20 ~ 25 min in a constant temperature incubator protected from light at 37°C. Finally, 6 μM of Propidium Iodide (PI) was added and the incubation was continued under dark conditions for 5 min. Afterwards, the cells were washed three times with PBS to remove excess staining reagent.

Results

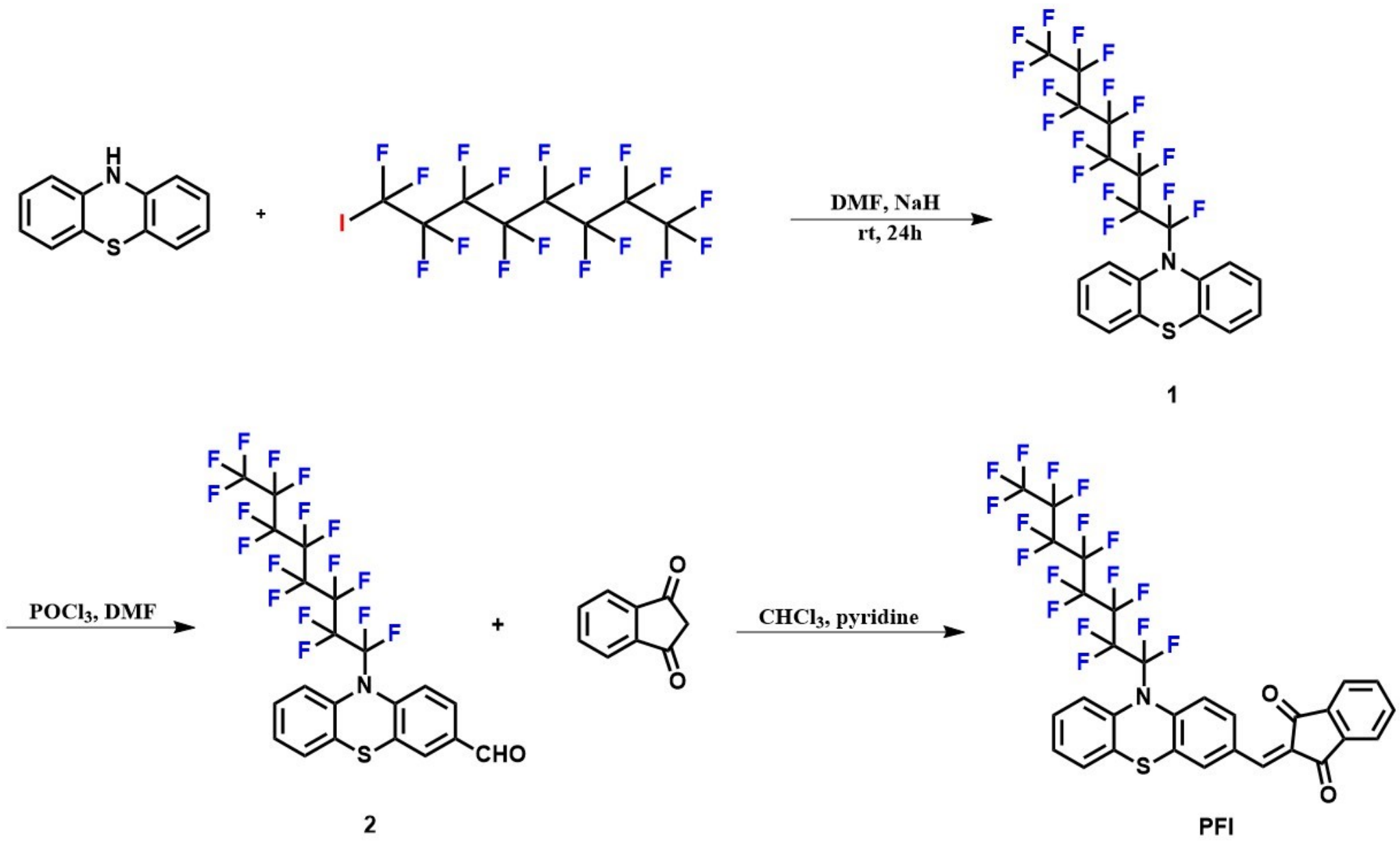

Scheme S1 (**A**) Synthetic routes of compound 1, compound 2 and targeted compound (PFI).

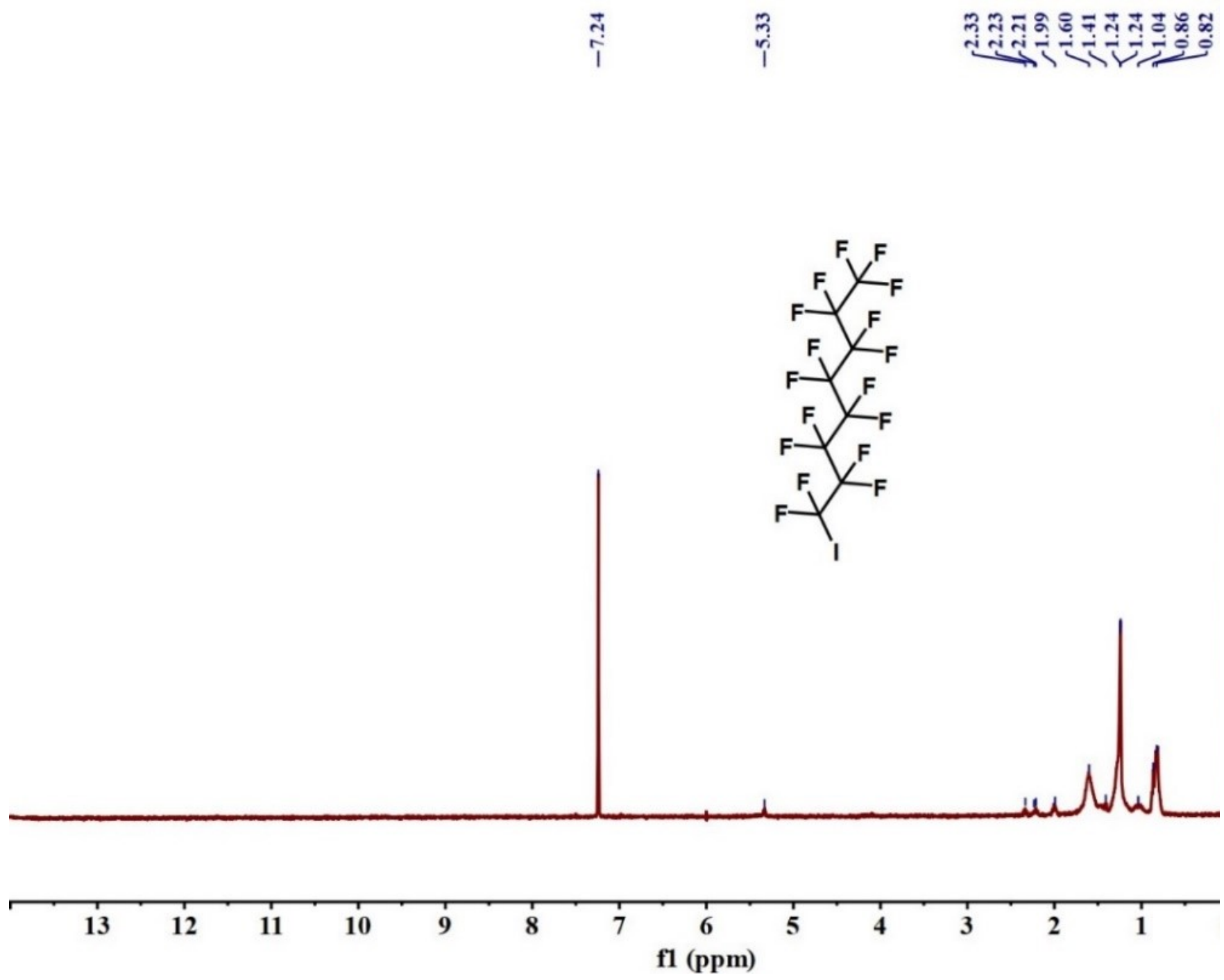

Fig. S1 ^{1}H NMR spectrum of perfluoro-n-Octyl Iodide.

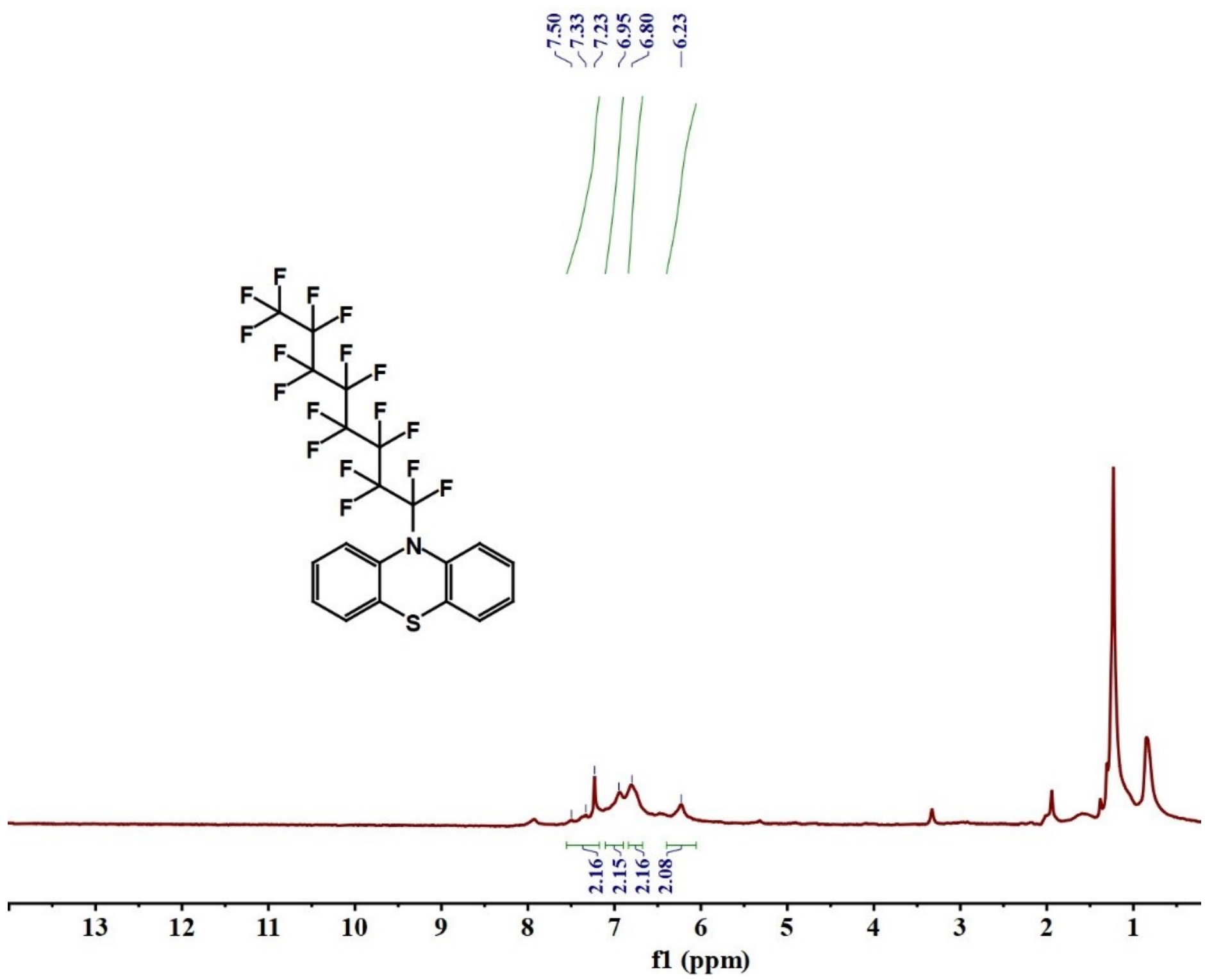

Fig. S2 ^{1}H NMR spectrum of compound 1.

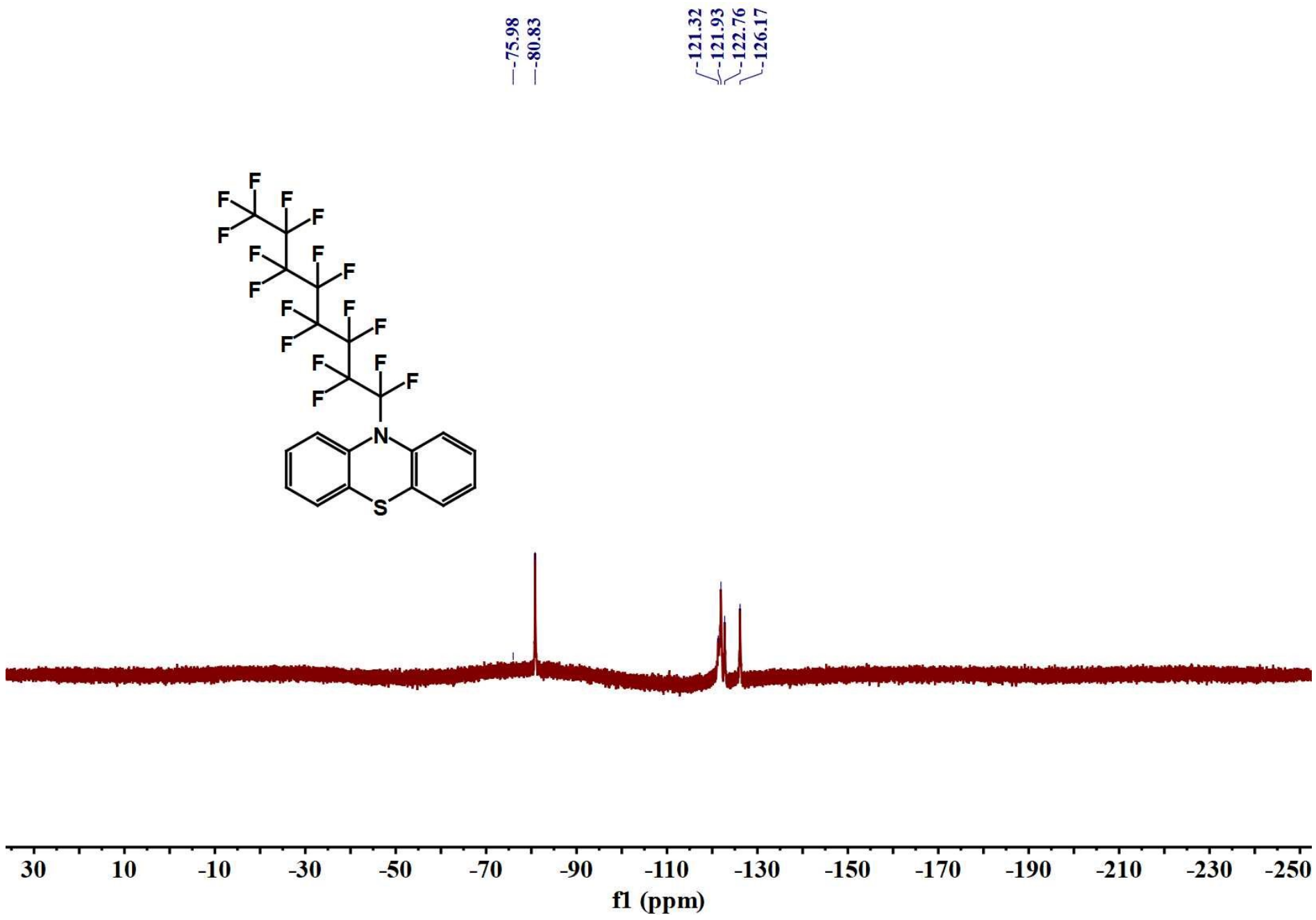

Fig. S3 ^{19}F NMR spectrum of compound 1.

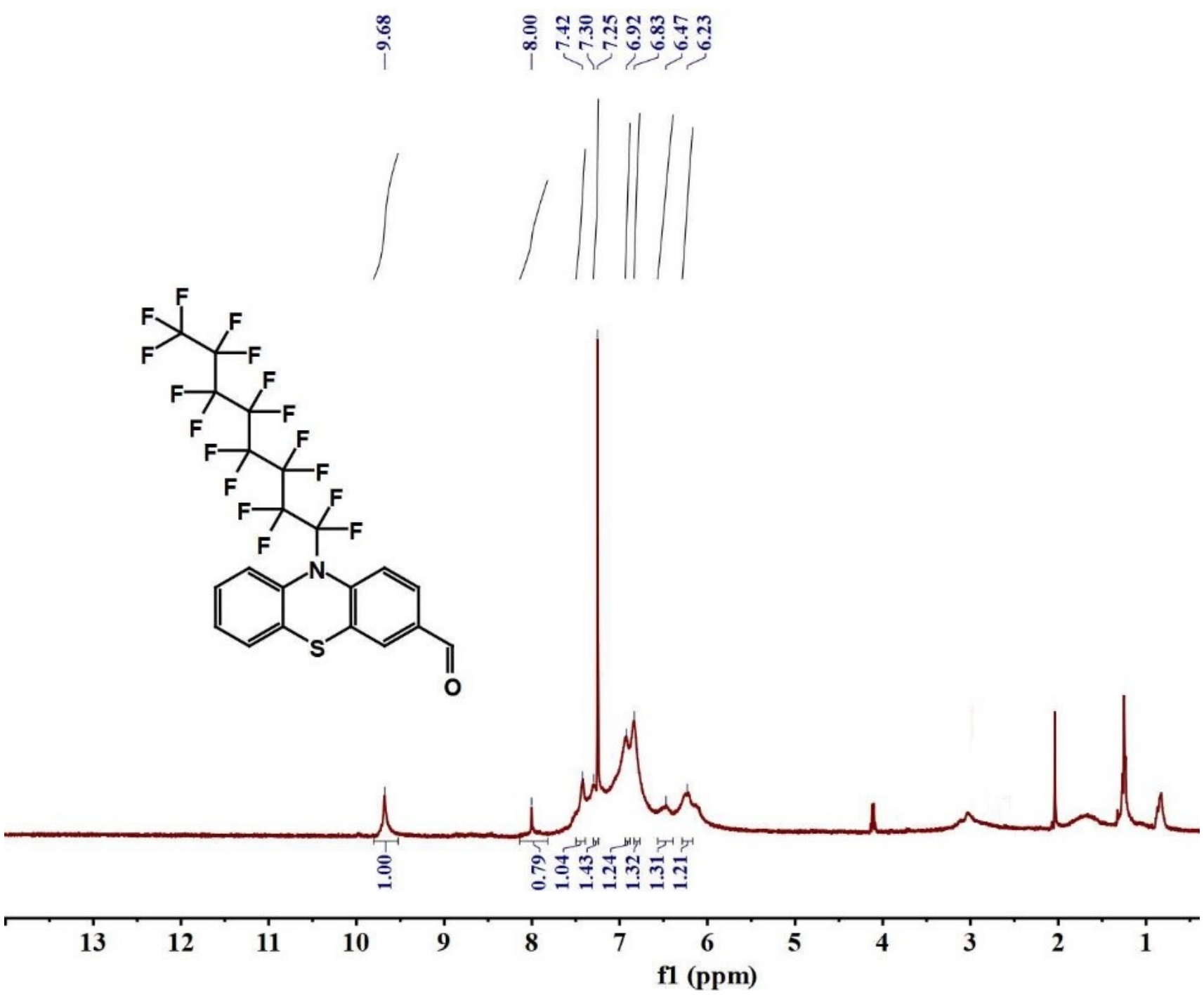

Fig. S4 ^{1}H NMR spectrum of compound 2.

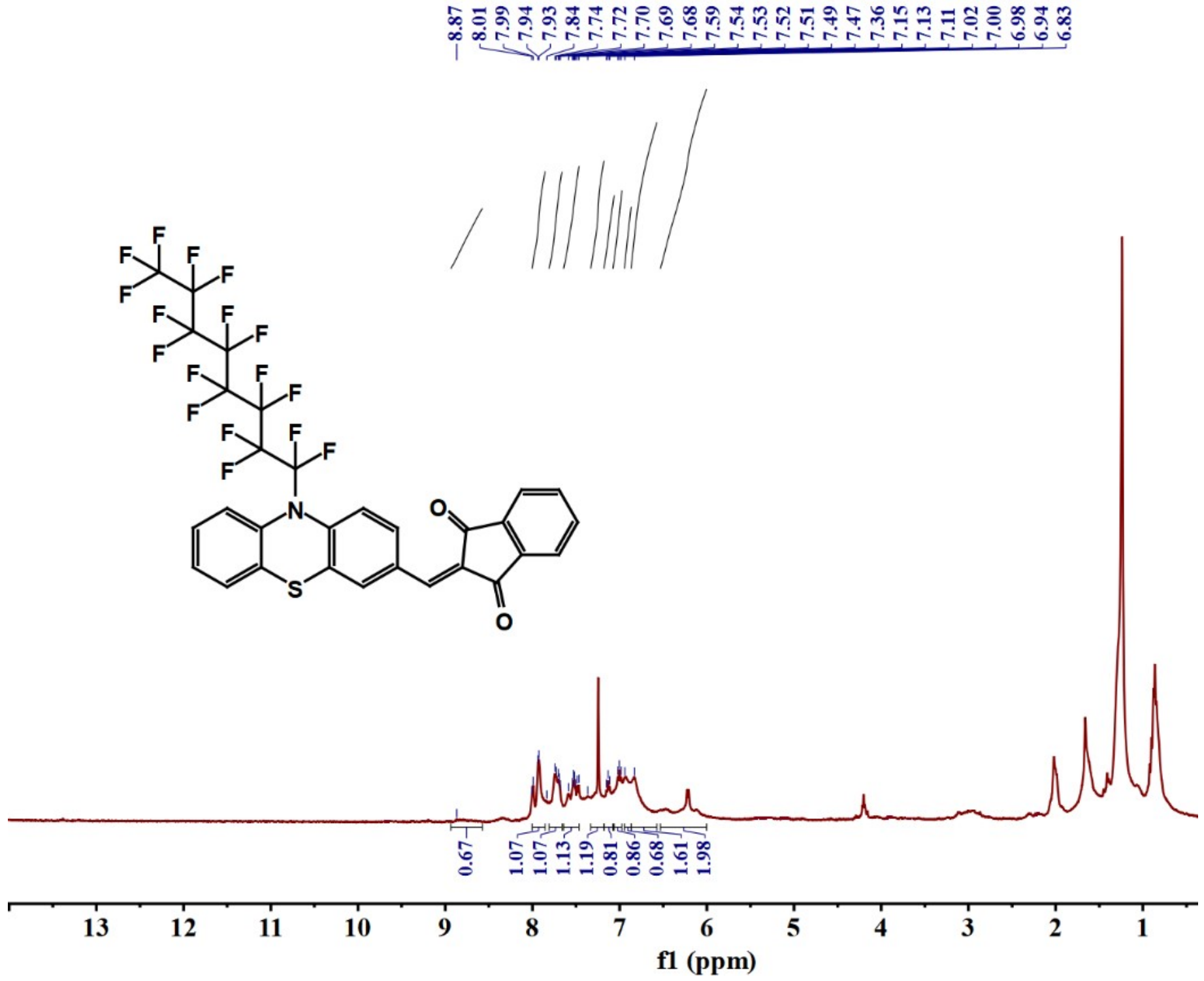

Fig. S5 ^{1}H NMR spectrum of PFI.

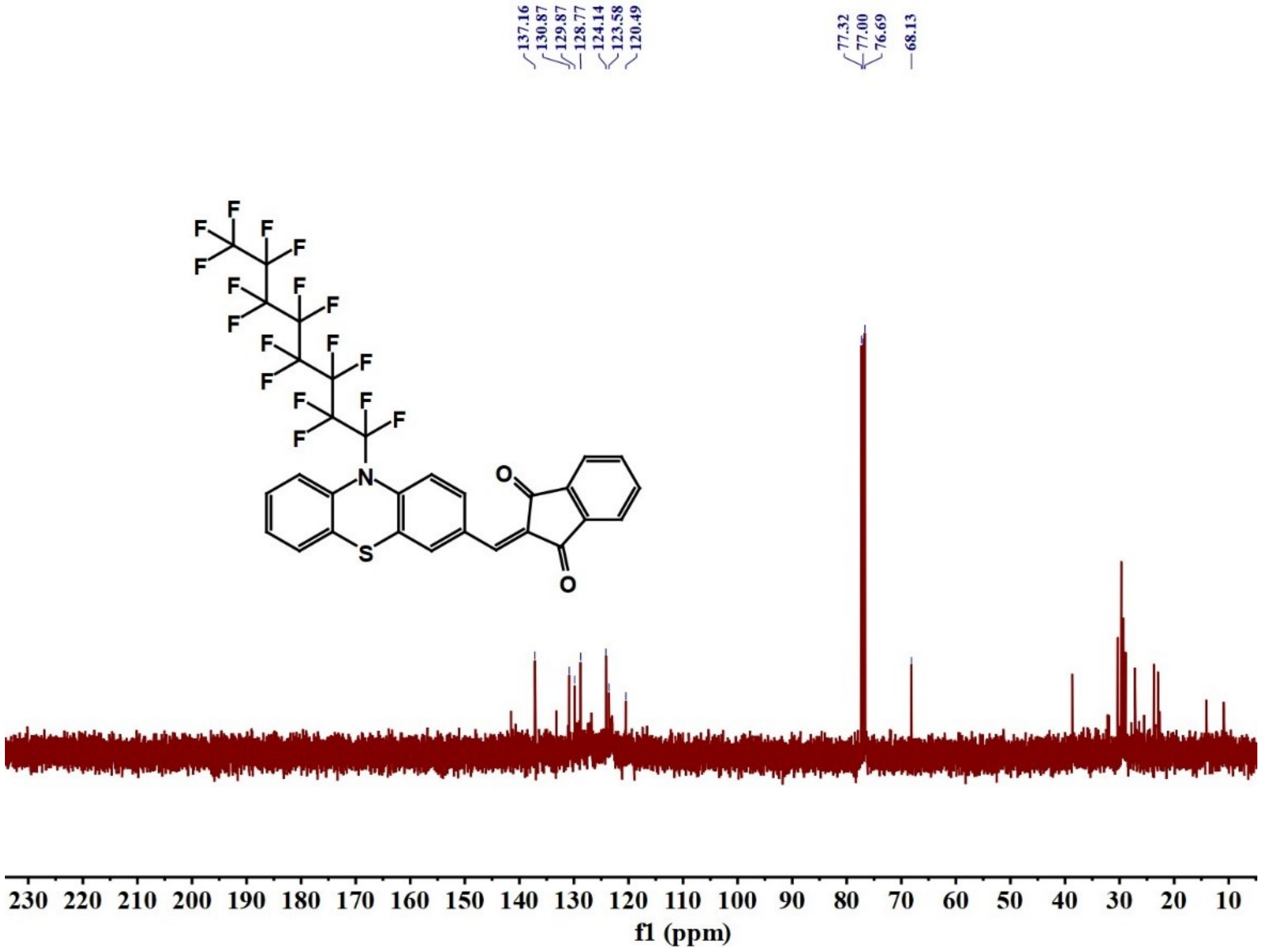

Fig. S6 ^{13}C NMR spectrum of PFI.

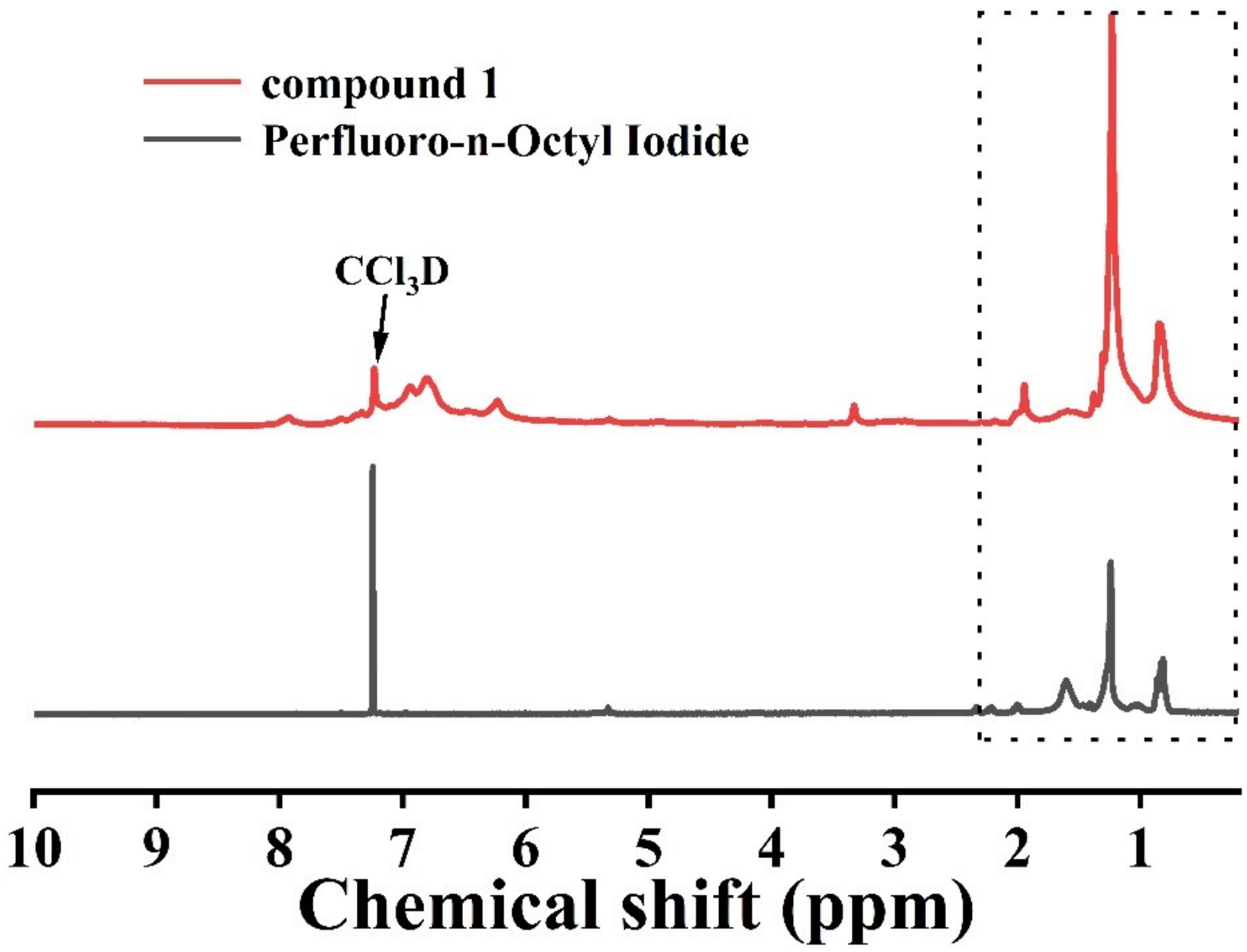

Fig. S7 ^{1}H NMR spectra of Perfluoro-n-Octyl Iodide and Compound 1.

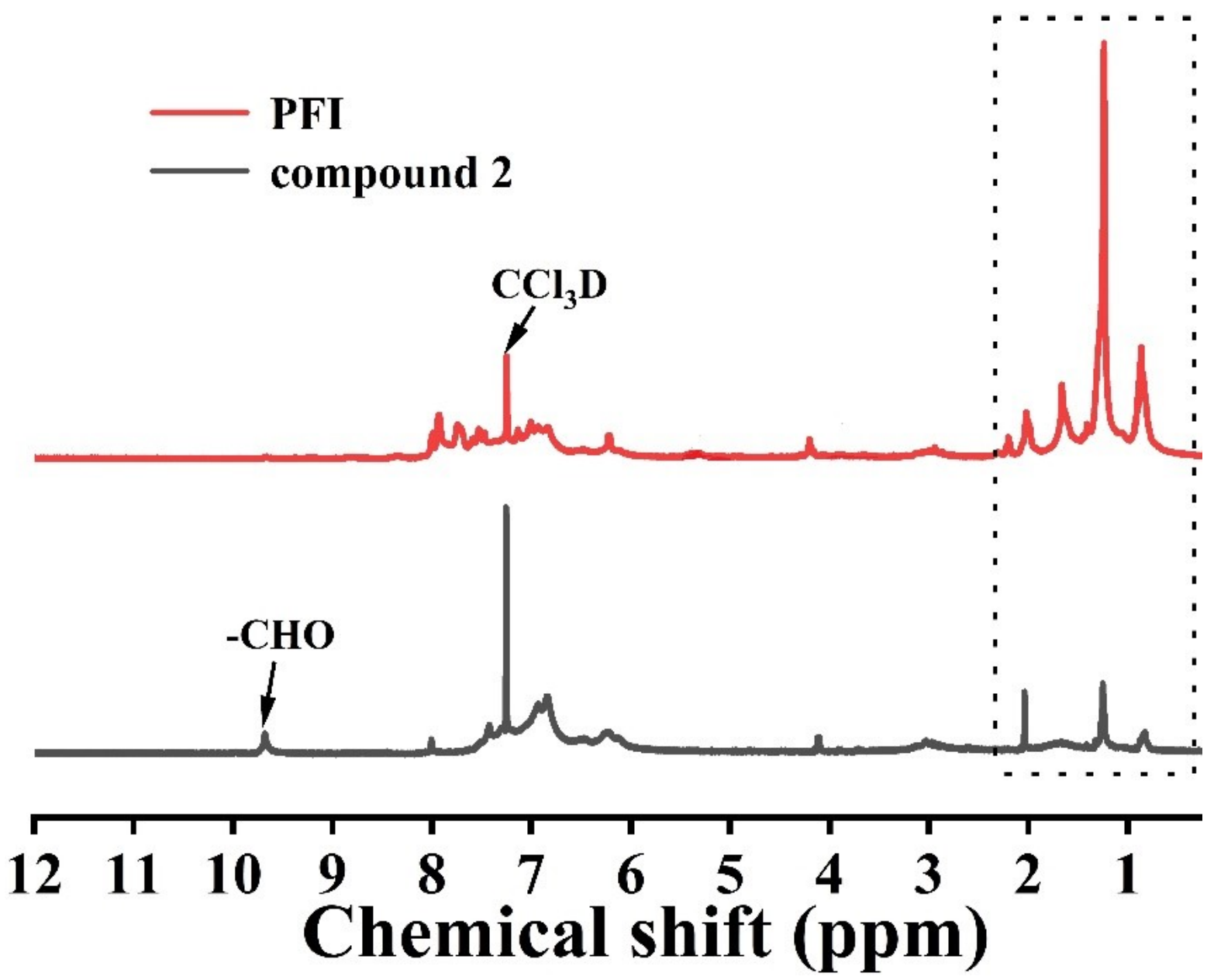

Fig. S8 ^{1}H NMR spectra of Compound 2 and PFI.

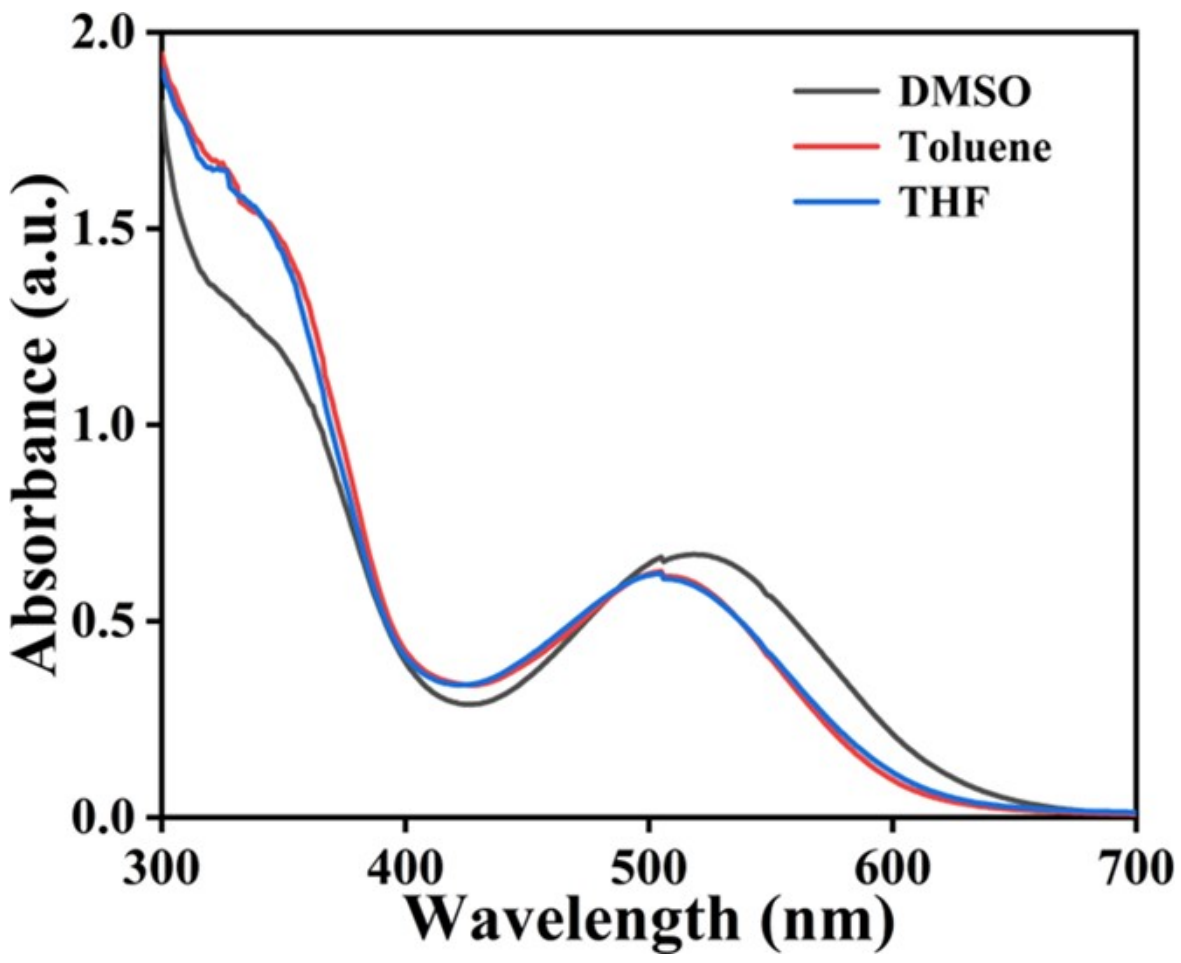

Fig. S9 UV absorption spectra of PFI in different solvents.

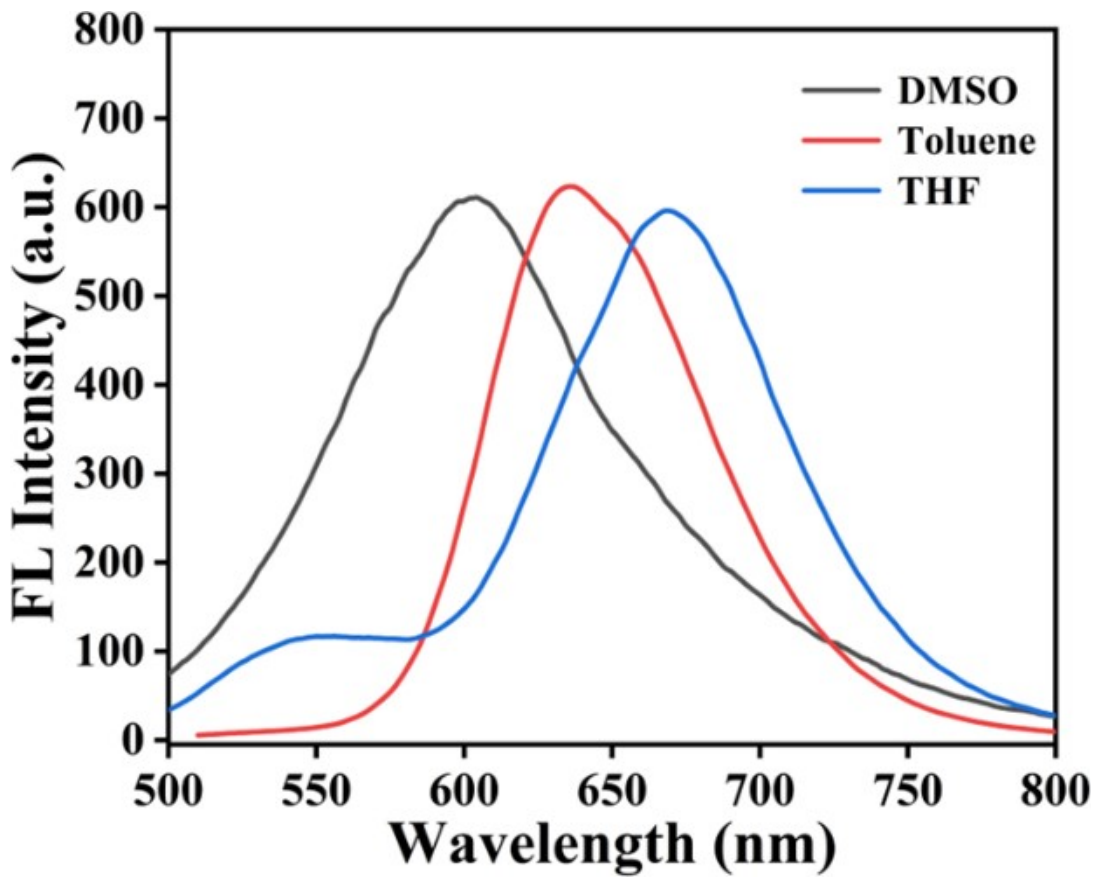

Fig. S10 Fluorescence emission spectra of PFI in different solvents.

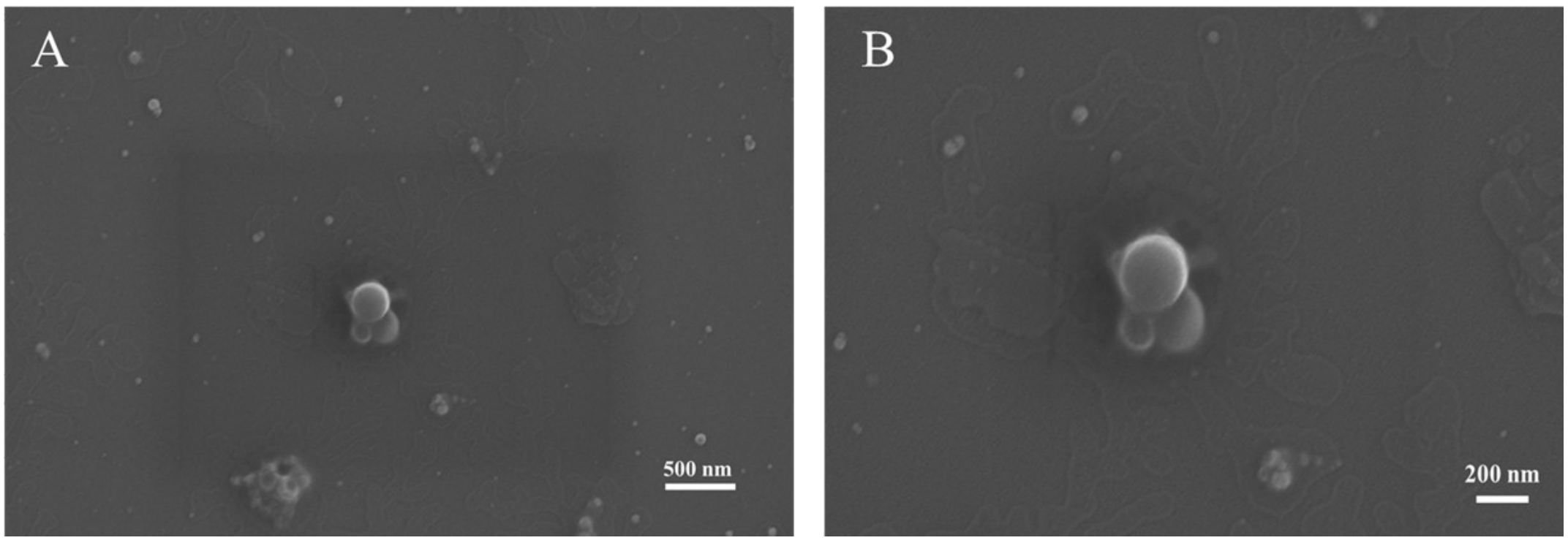

Fig. S11 SEM images of PFI NPs. (**A**) Low-magnification SEM image showing the overall morphology and distribution of PFI NPs. (**B**) High-magnification SEM image revealing the well-defined, near-spherical shape of individual nanoparticles.

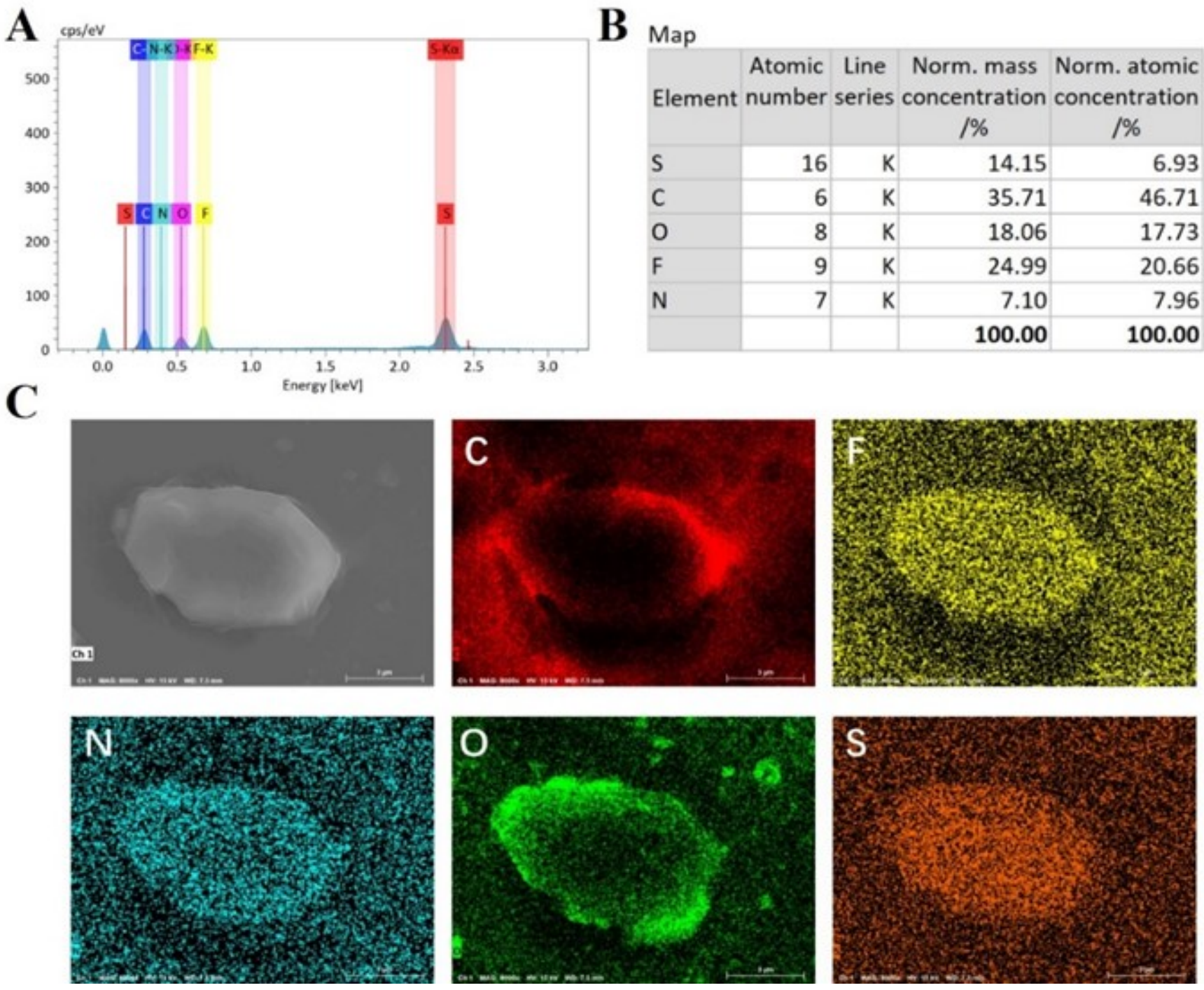

Element	Atomic number	Line series	Norm. mass concentration /%	Norm. atomic concentration /%
S	16	K	14.15	6.93
C	6	K	35.71	46.71
O	8	K	18.06	17.73
F	9	K	24.99	20.66
N	7	K	7.10	7.96
			100.00	100.00

Fig. S12 Elemental composition and distribution of PFI NPs characterized by energy-dispersive X-ray spectroscopy (EDS). (**A**) EDS spectrum showing characteristic peaks of carbon (C), nitrogen (N), oxygen (O), fluorine (F), and sulfur (S). (**B**) Quantitative elemental analysis table listing normalized mass and atomic concentrations. (**C**) Corresponding EDS mapping images of C, N, O, F, and S, demonstrating homogeneous distribution of all elements across the nanoparticle assembly.

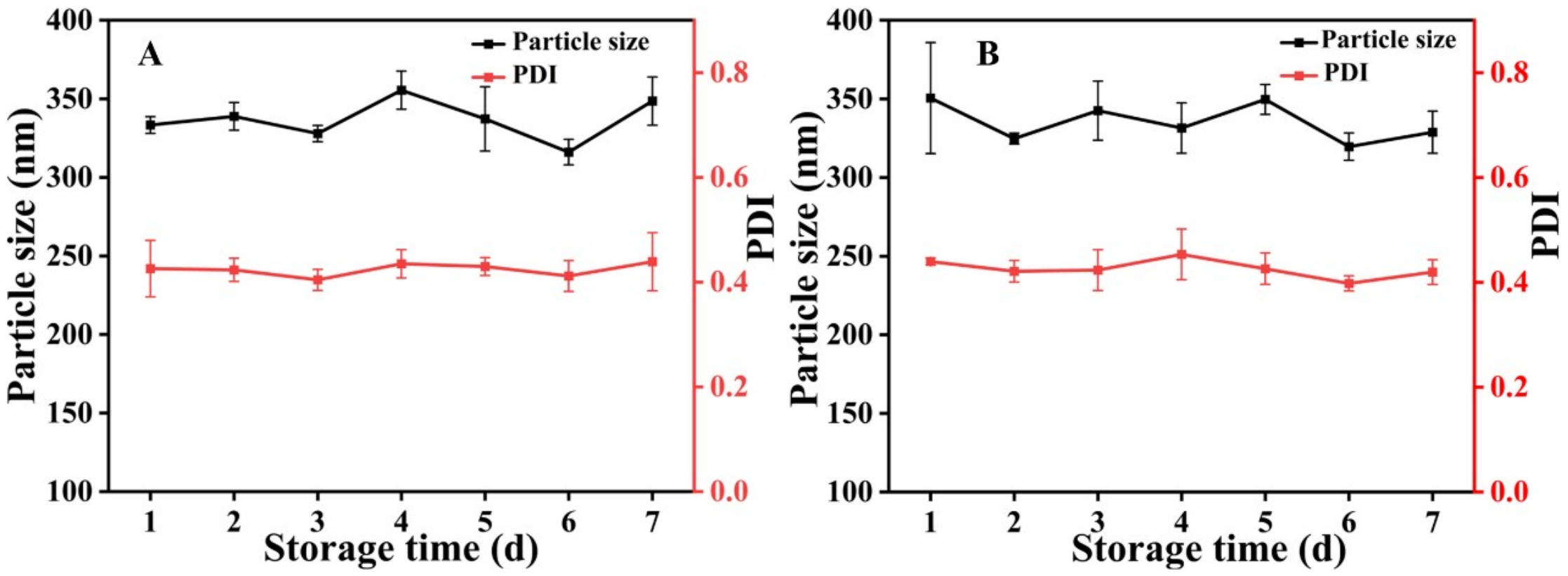

Fig. S13 Changes in particle size and PDI of PFI NPs during a 7-day storage period: (A) in water, (B) in PBS.

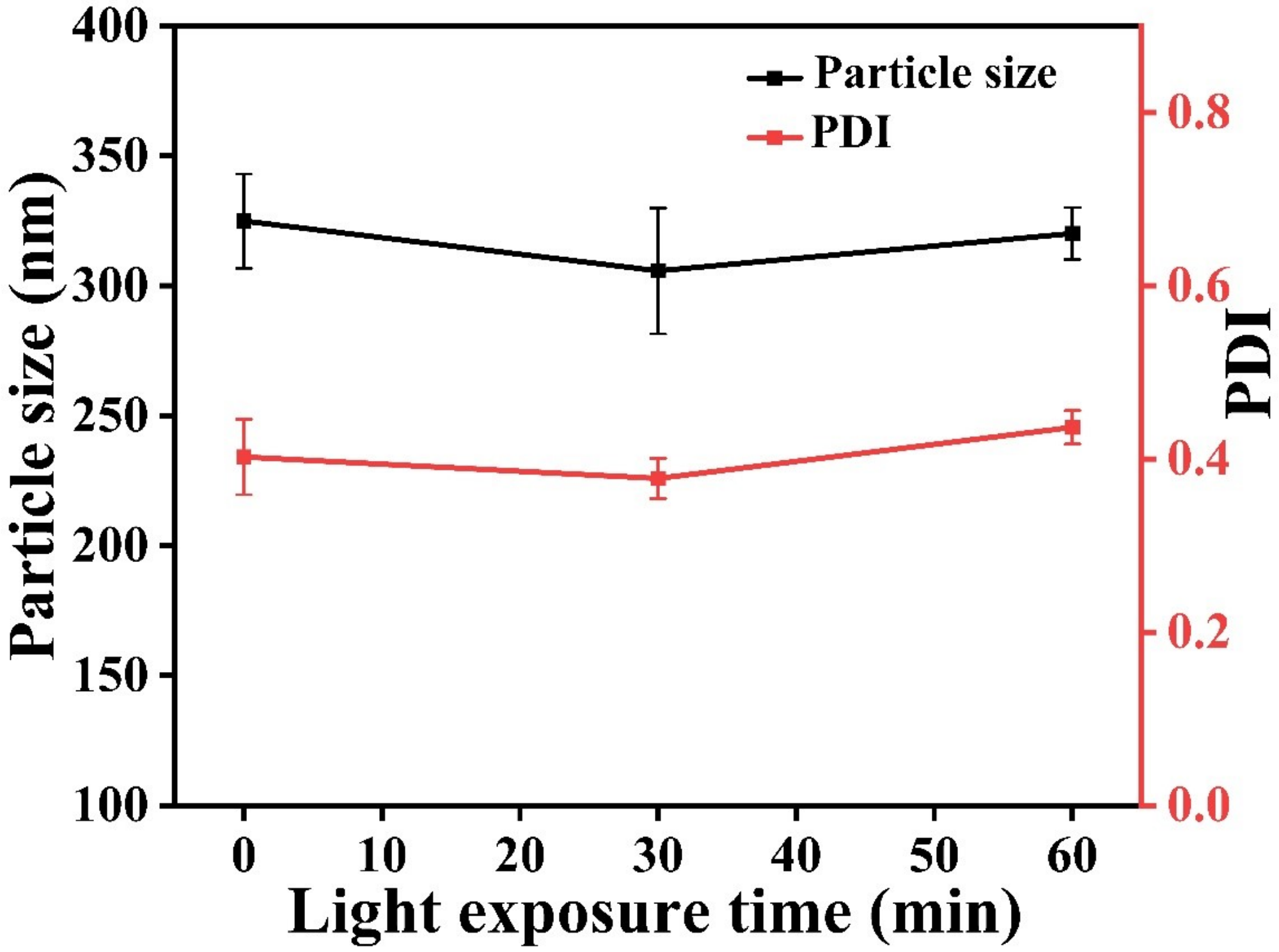

Fig. S14 Changes in particle size and PDI of PFI NPs after 0, 30, and 60 minutes of Light Exposure; white light irradiation (50 mW/cm^2).

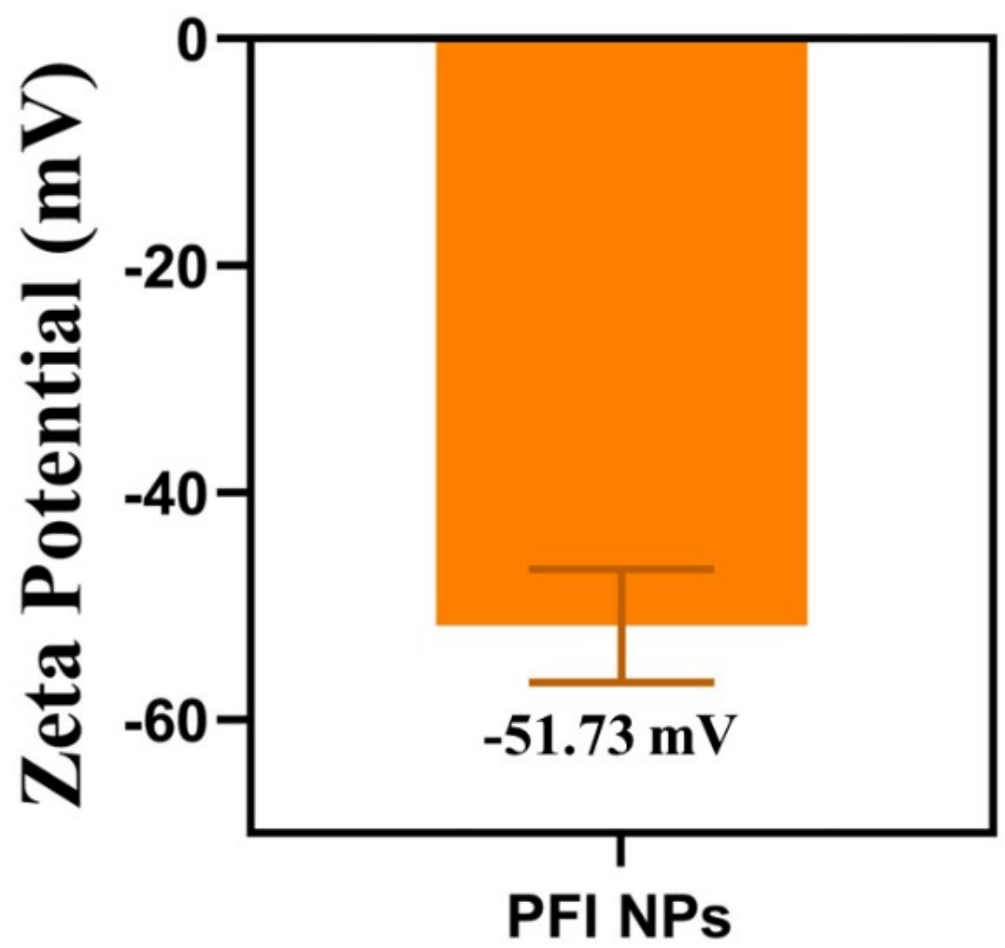

Fig. S15 Zeta potential measurement of PFI NPs.

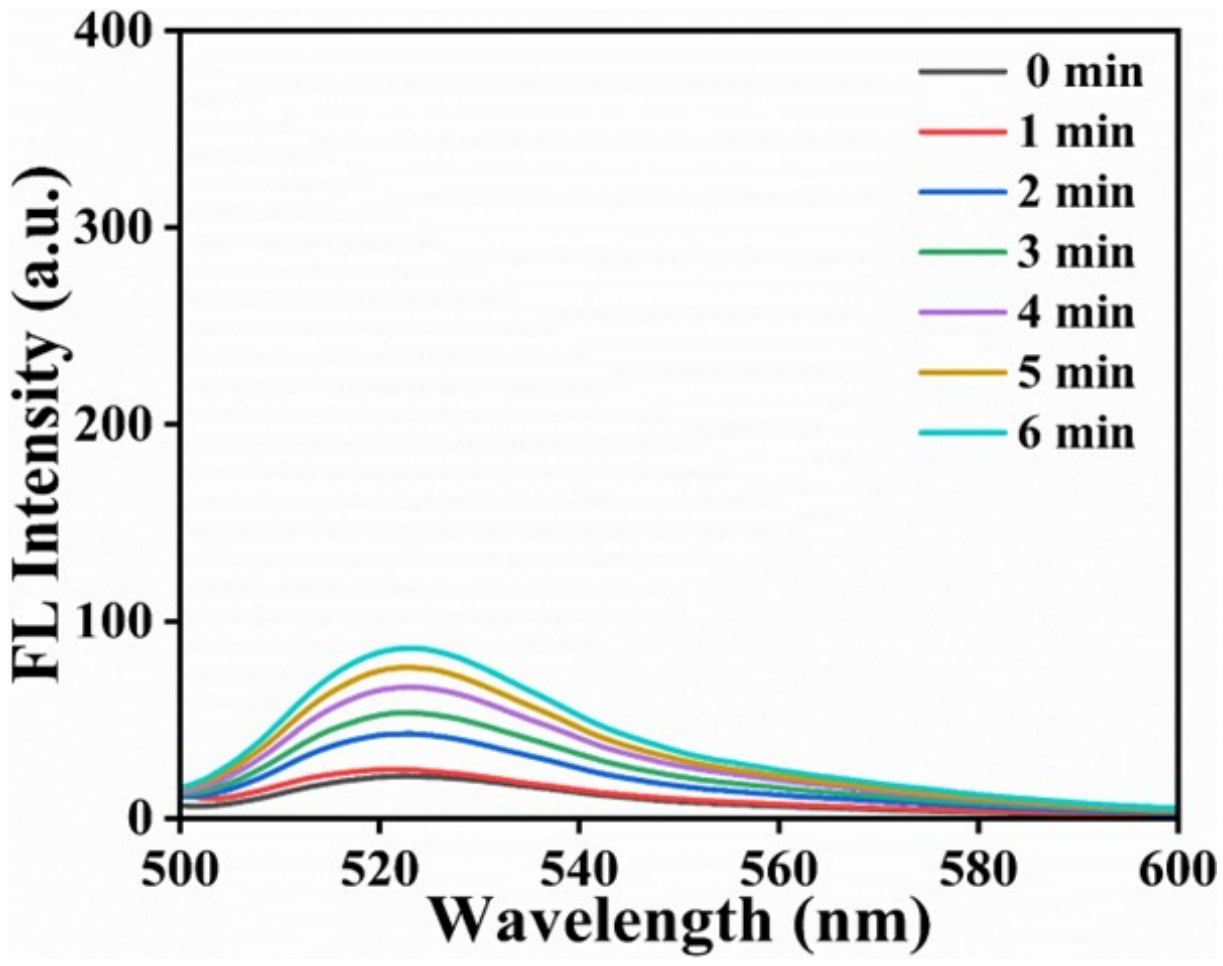

Fig. S16 PL spectra of DCFH-DA of blank after light irradiation. [excitation wavelength: 488 nm, emission wavelength: 500-650 nm, excitation bandwidth: 10 nm, emission bandwidth: 10 nm, PMT: 400 V]; white light irradiation (50 mW/cm^2).

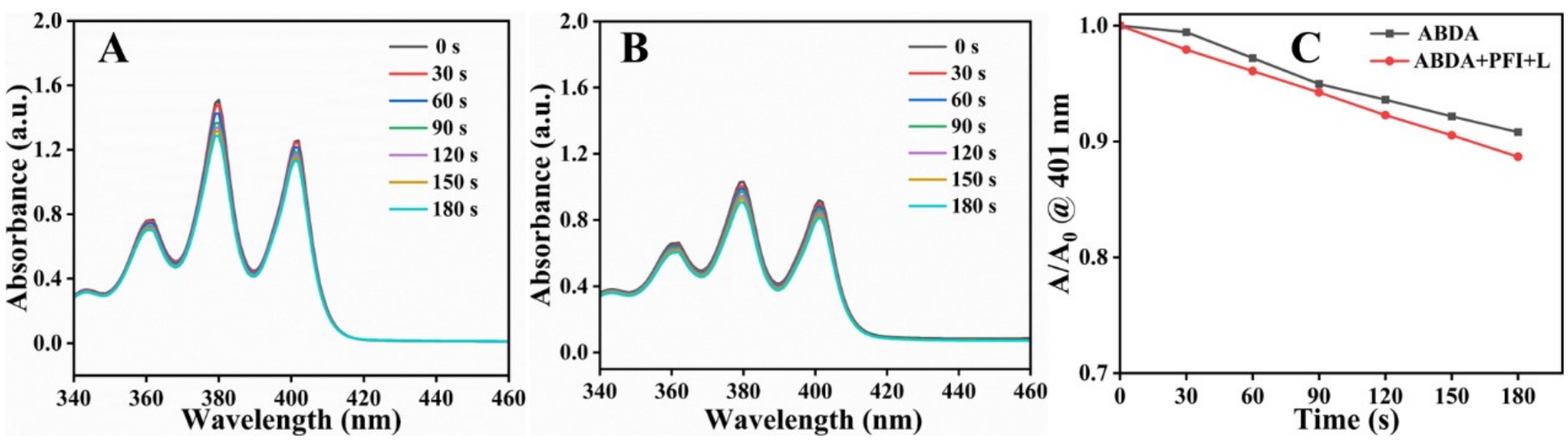

Fig. S17 The UV-visible absorption spectra of ABDA after light irradiation in the present of PFI. (**A**) Blank, (**B**) PFI, (**C**)PFI at the initial time of 0-180 s. [PS] = 1 mg/mL, [ABDA] = 4 mM; white light irradiation (50 mW/cm^2).

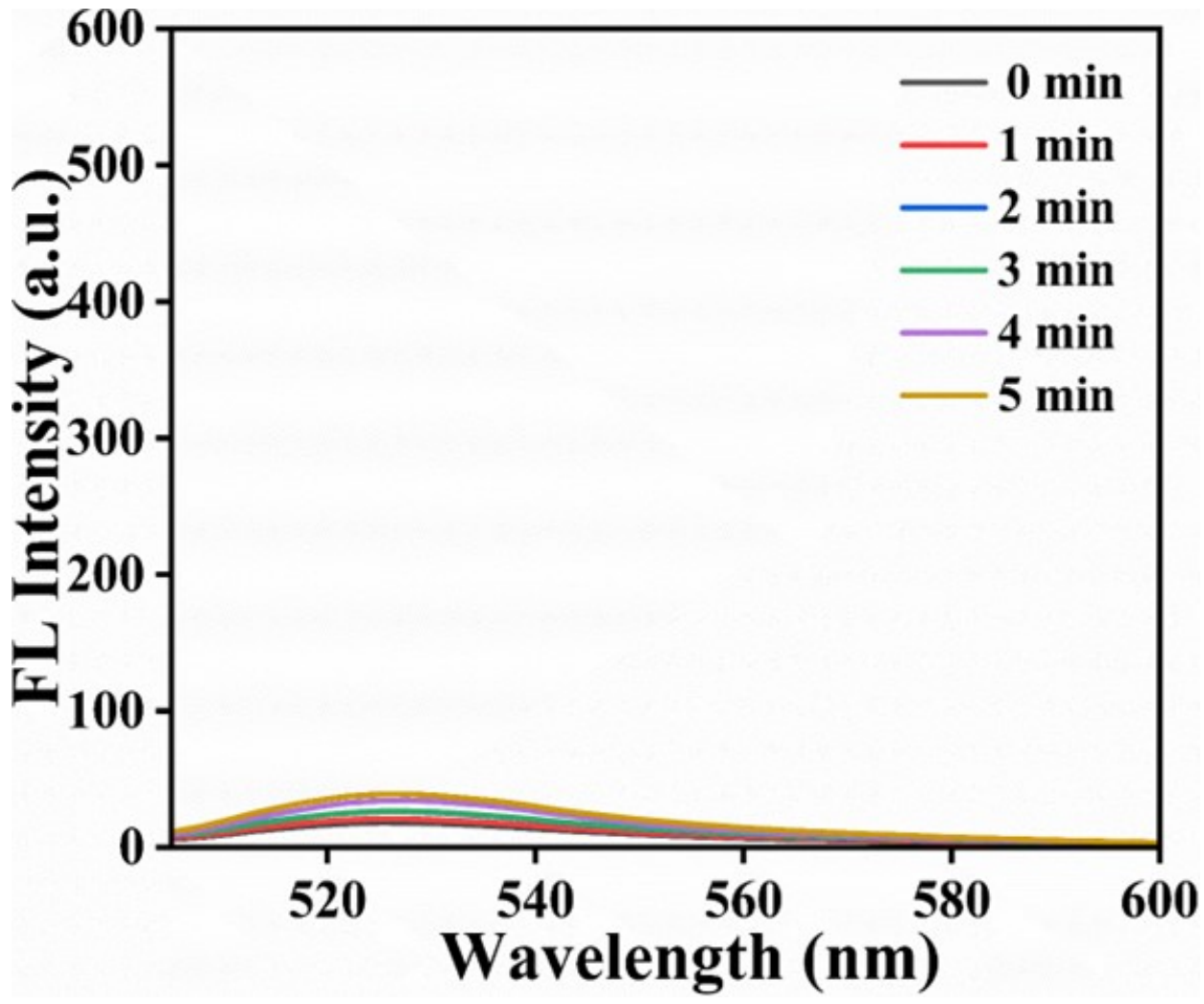

Fig. S18 PL spectra of DHR123 of blank after light irradiation. [Excitation wavelength: 488 nm, emission wavelength: 500-650 nm, PMT: 400V, excitation bandwidth: 10 nm, emission bandwidth: 10 nm. [DHR123] = 1 μM; white light irradiation (50 mW/cm^2)].

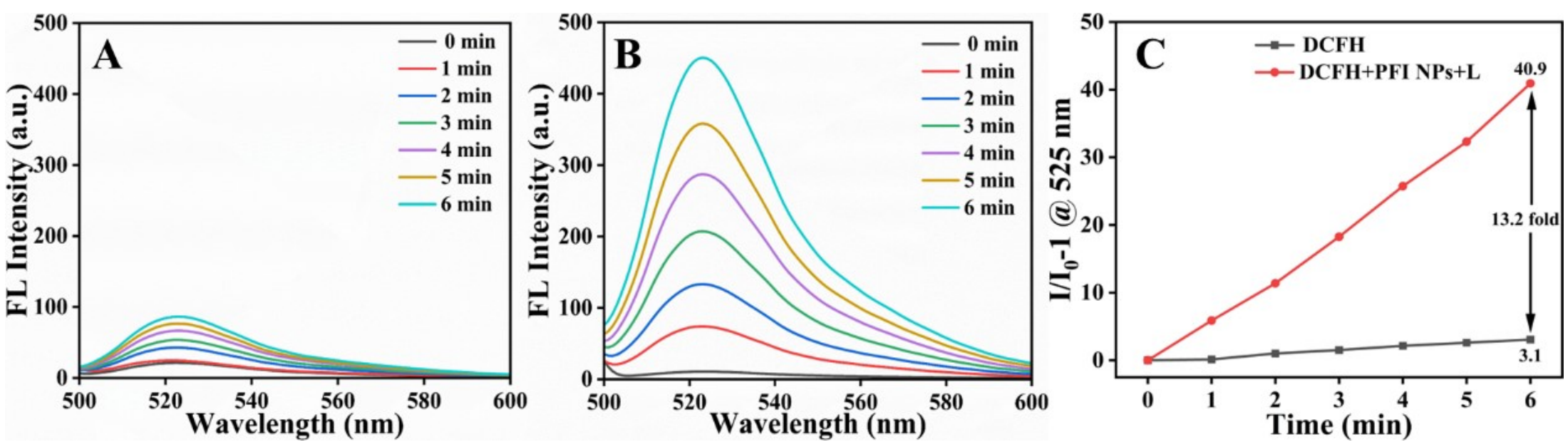

Fig. S19 PL spectra of DCFH in the absence and presence of PFI NPs under irradiation with different time in DMSO/PBS (v/v=1/99): (**A**) Blank, (**B**) PFI NPs, (**C**) Fluorescence intensity ratios of blank and PFI NPs at 525 nm at 6 min. Excitation wavelength: 488 nm, emission wavelength: 500-650 nm, PMT: 400 V, excitation bandwidth: 10 nm, emission bandwidth: 10 nm. [PS] = 200 μg/mL, [DCFH] = 1 μM; white light irradiation (50 mW/cm^2).

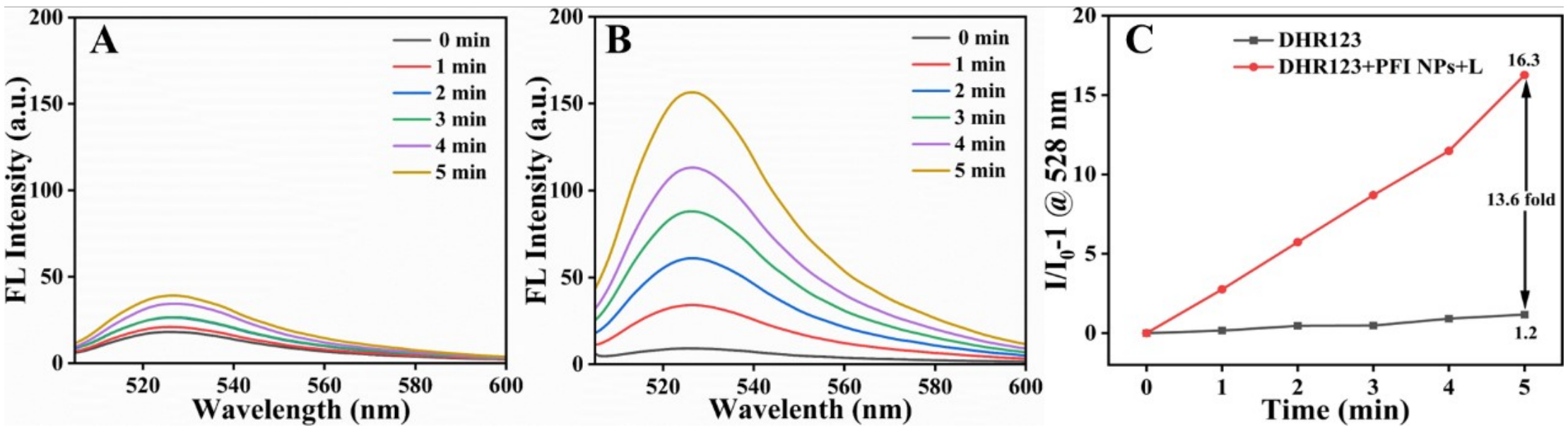

Fig. S20 PL spectra of DHR123 in the absence and presence of PS NPs under irradiation with different time in DMSO/PBS (v/v=1/99): (**A**) Blank, (**B**) PFI NPs, (**C**) Fluorescence intensity ratio of blank and PFI NPs at 528 nm at 5 min. Excitation wavelength: 488 nm, Emission wavelength:500-650 nm, PMT: 400 V, excitation bandwidth: 10 nm, emission bandwidth: 10 nm. [PS] =200 μg/mL, [DHR123] = 1 μM; white light irradiation (50 mW/cm^2).

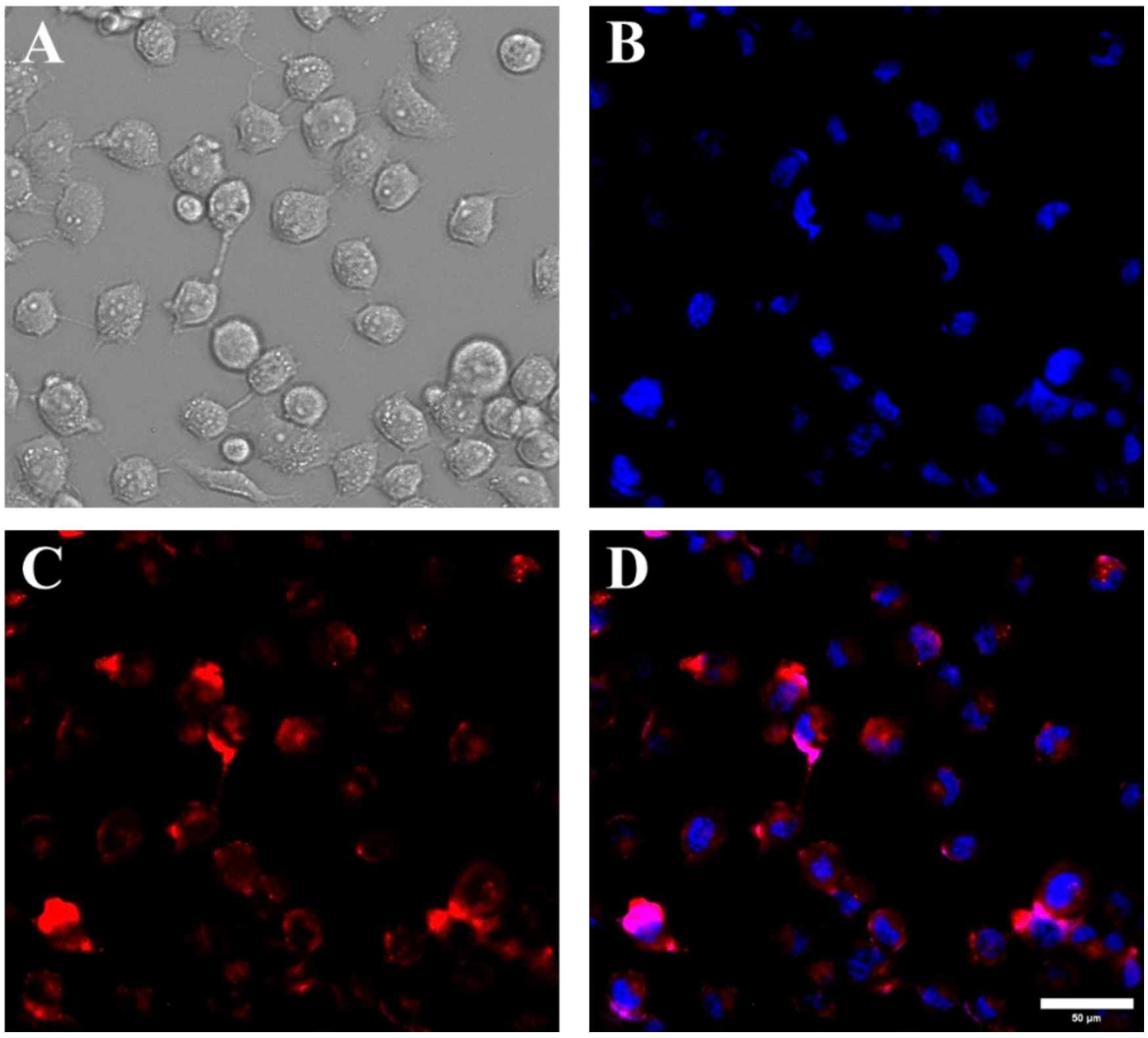

Fig. S21 CLSM images of MDA-Mb-231 cancer cells, (**A**) bright field, (**B**) fluorescence image of cells stained with DAPI, (**C**) fluorescence image of cells after incubation with PFI NPs, (**D**) superimposed image, scale bar = 50 μm.

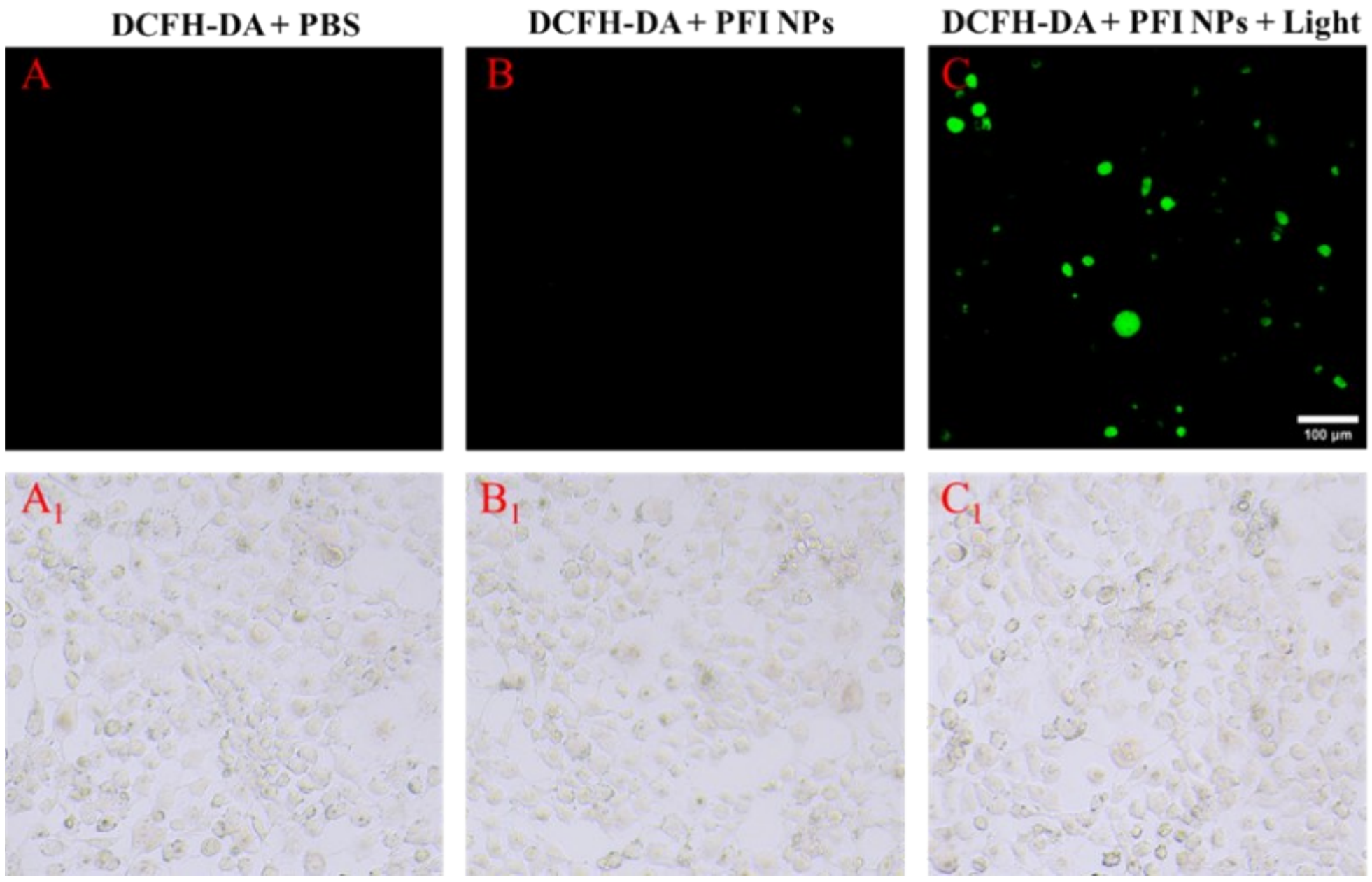

Fig. S22 Fluorescence microscopy images of ROS generation of PFI NPs (30 μg/mL) in MDA-MB-231 cells under light irradiation (5 mW/cm^2) for 3 min by using DCFH-DA indicator (1×10^{-5} M), scale bar: 100 μm.

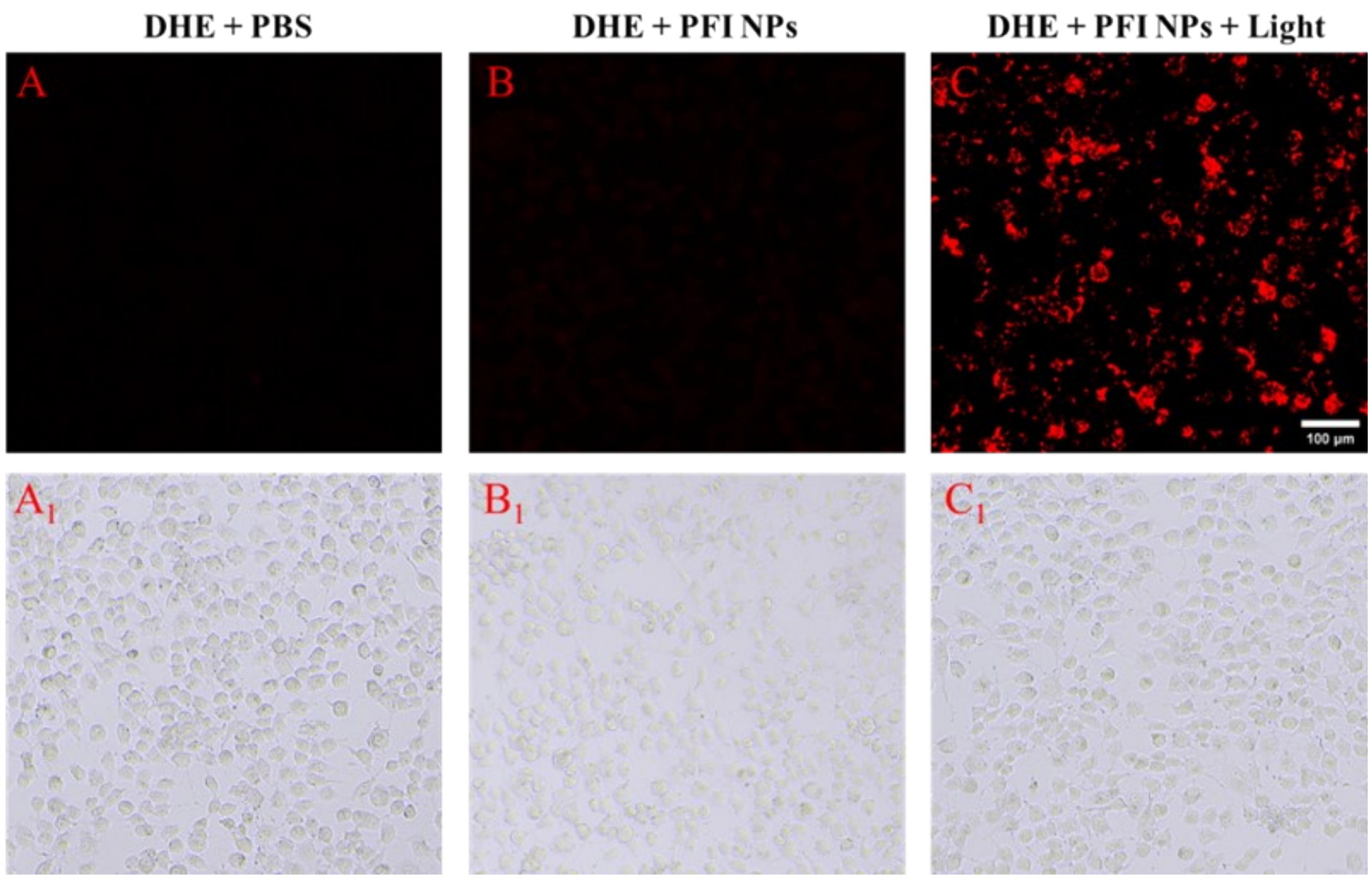

Fig. S23 Fluorescence microscopy images of free radicals of PFI NPs (30 μg/mL) in MDA-MB-231 cells under light irradiation (5 mW/cm^2) for 3 min by using DHE (1×10^{-5} M), scale bar: 100 μm.

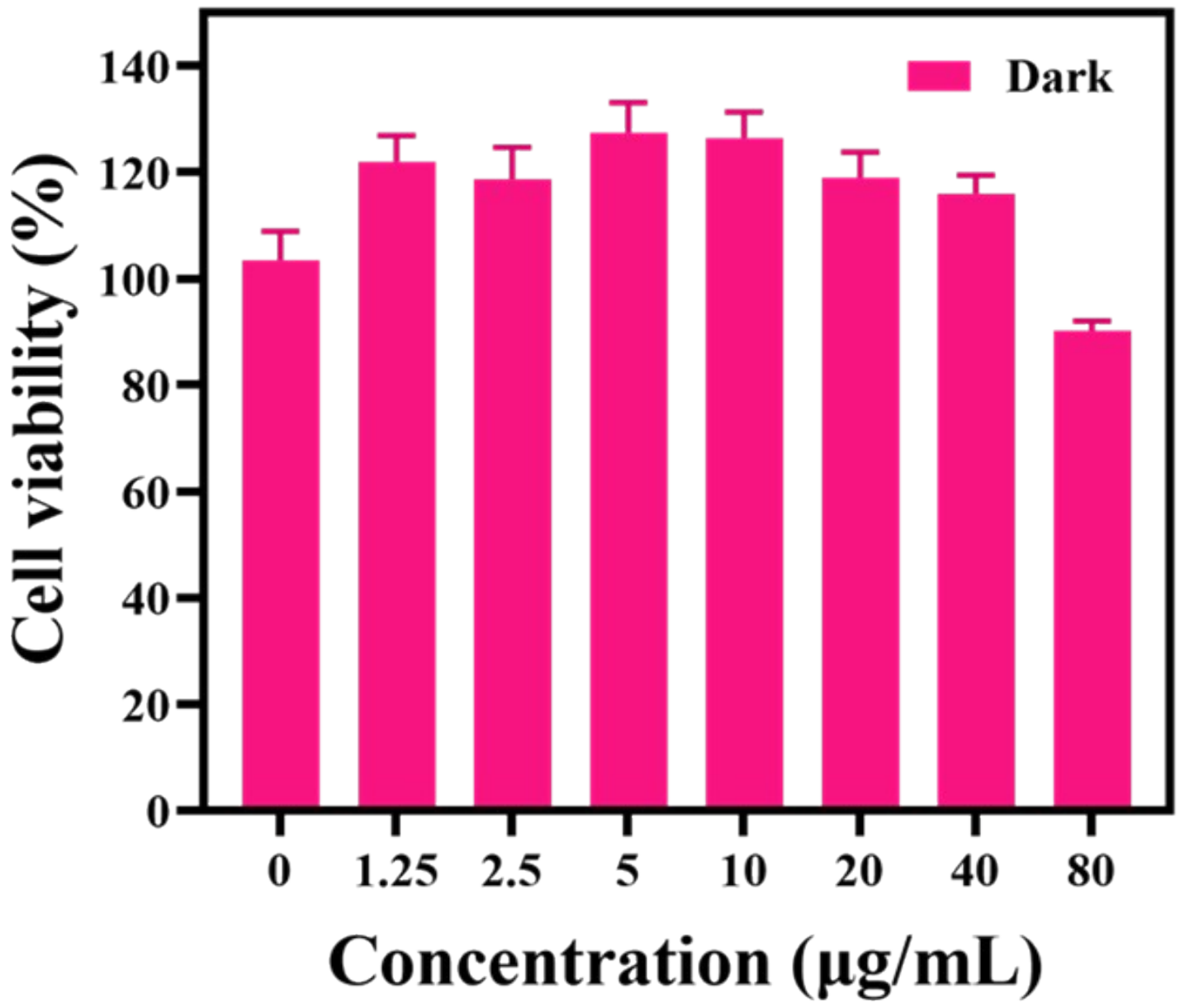

Fig. S24 Dark cell viability after co-incubation of PFI NPs with MDA-MB-231 cells at different concentrations.

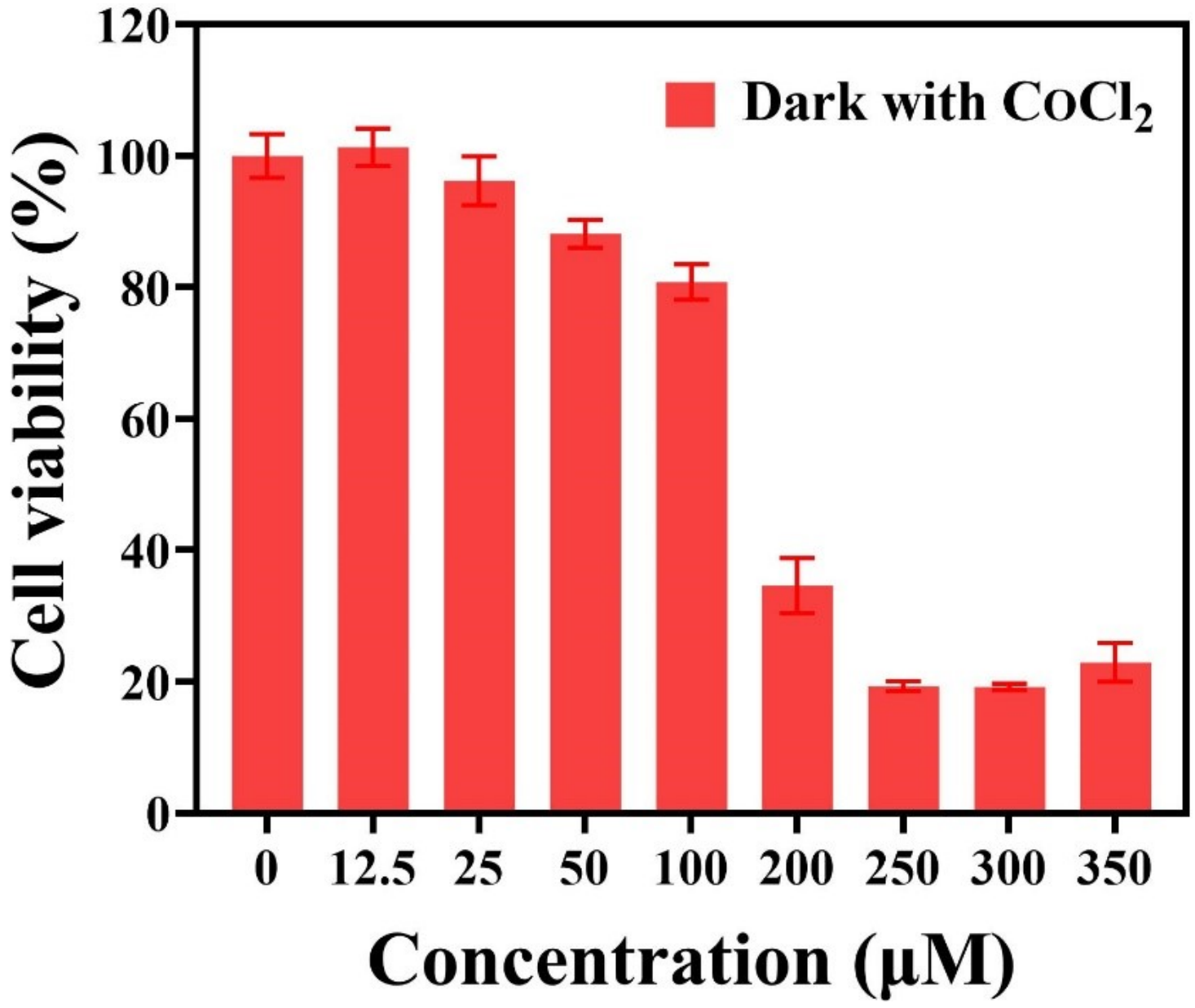

Fig. S25 Dark cell toxicity after co-incubation of cobalt chloride ($CoCl_2$) with MDA-MB-231 cells at different concentrations.

References

1 Tang, Y. *et al.* Salidroside attenuates CoCl2-simulated hypoxia injury in PC12 cells partly by mitochondrial protection. *European journal of pharmacology* **912**, 174617 (2021).

2 Rana, N. K., Singh, P. & Koch, B. CoCl2 simulated hypoxia induce cell proliferation and alter the expression pattern of hypoxia associated genes involved in angiogenesis and apoptosis. *Biological research* **52**, 12 (2019).

3 Muñoz-Sánchez, J. & Chánez-Cárdenas, M. E. The use of cobalt chloride as a chemical hypoxia model. *Journal of Applied Toxicology* **39**, 556-570 (2019).
